# Blue Light Sensing BlsA-Mediated Modulation of Meropenem Resistance and Biofilm Formation in Acinetobacter baumannii

**DOI:** 10.1128/msystems.00897-22

**Published:** 2023-01-09

**Authors:** Jihye Yang, Sohyeon Yun, Woojun Park

**Affiliations:** a Laboratory of Molecular Environmental Microbiology, Department of Environmental Science and Ecological Engineering, Korea University, Seoul, Republic of Korea; University of Wisconsin-Madison

**Keywords:** multidrug-resistance, BLUF domain, bacterial two-hybrid system, OmpA, membrane permeability

## Abstract

The presence or absence of BlsA, a protein with a blue light-sensing flavin domain in the genomes of Acinetobacter species has aroused curiosity about its roles in the regulation of bacterial lifestyle under light. Genomic and transcriptomic analyses revealed the loss of BlsA in several multidrug-resistant (MDR) A. baumannii strains as well as the light-mediated induction of *blsA*, along with a possible BlsA-interacting partner BipA. Their direct *in vivo* interactions were verified using a bacterial two-hybrid system. The results demonstrated that the C-terminal region of BipA could bind to the C-terminal residues of BlsA under blue light at 23°C but not at 37°C. Genetic manipulations of *blsA* and *bipA* revealed that the coexistence of BlsA and BipA was required to induce the light-dependent expression of *ompA* in A. baumannii ATCC 17978 at 23°C. The same phenomenon occurred in the BlsA-deficient MDR strain in our functional complementation assay; however, the underlying molecular mechanism remains poorly understood. BlsA-modulated amounts of OmpA, the most abundant porin, in the outer membrane affected the membrane integrity and permeability of small molecules. Dark conditions or the deletion of *ompA* made the membrane more permeable to lipophilic ethidium bromide (EtBr) but not to meropenem. Interestingly, light illumination and low temperature conditions made the cells more sensitive to meropenem; however, this bactericidal effect was not noted in the *blsA* mutant or in the BlsA-deficient MDR strains. Light-mediated cell death and the reduction of biofilm formation at 23°C were abolished in the *blsA* mutant strain, suggesting multifaceted roles of BlsA in A. baumannii strains.

**IMPORTANCE** Little is known about the functional roles of BlsA and its interacting partners in Acinetobacter species. Intriguingly, no BlsA homolog was found in several clinical isolates, suggesting that BlsA was not required inside the host because of the lack of blue light and the warm temperature conditions. As many chromophore-harboring proteins interact with various partners to control light-dependent cellular behaviors, the maintenance of *blsA* in the genomes of many Acinetobacter species during their evolution may be beneficial when fluctuations occur in two important environmental factors: light and temperature. Our study is the first to report the novel protein partner of BlsA, namely, BipA, and its contribution to multiple phenotypic changes, including meropenem resistance and biofilm formation. Rapid physiological acclimation to changing light or temperature conditions may be possible in the presence of the light-sensing BlsA protein, which may have more interacting partners than expected.

## INTRODUCTION

Light perception in nonphototrophic bacteria using chromophore-harboring proteins (e.g., blue light-using flavin [BLUF], rhodopsin, and phytochromes) has been noted in several environmental bacteria, such as Escherichia coli and Bacillus subtilis, as well as in clinically important pathogens, such as Acinetobacter baumannii and Klebsiella pneumoniae (1–4). Although concrete molecular and ecological data regarding these photoreceptor proteins remain unknown, the light-dependent modulation of bacterial physiology and behaviors (e.g., biofilm formation, iron homeostasis, and antibiotic resistance) has been experimentally elucidated in the aforementioned bacterial groups (5–7). The BLUF domain-mediated, light-sensing protein YcgF, which functions as a direct antagonist of the YcgE repressor, was found to be linked to the production of capsular colanic acid and fimbriae through the release of YcgE from the DNA operator in E. coli, which could occur only at temperatures below 28°C because of the low temperature-induced expression of the YcgF/YcgE system (8, 9). Light-induced structural changes in B. subtilis YtvA that possessed a flavin-based light-oxygen-voltage (LOV) domain were found to be involved in the activation of σ^B^, resulting in the protection of cellular macromolecules from light-generated reactive oxygen species (ROS) (10, 11). CryB of anoxygenic, phototrophic Rhodobacter sphaeroides acts as a proteobacterial cryptochrome and interacts with the BLUF-domain photoreceptor AppA, which controls the expression of photosynthetic genes by antagonizing the PpsR repressor (12).

Although multiple sets of different photoreceptors covering phytochromes, LOV domain proteins, and BLUF domain proteins are present in the genomes of phototrophic bacteria, including the purple bacterium R. sphaeroides, because of their roles in response to light-induced stresses and ROS defense (13), only one BLUF domain protein, designated BlsA, was present in A. baumannii ATCC 17978, A. nosocomialis, and Acinetobacter strain 5-2Ac02 (4, 14, 15). The physiological roles of BlsA, a blue light-sensing protein, in pathogenic bacteria remain vague. However, BlsA is likely involved in light-induced protein-protein interactions, presumably by the same mechanisms of the aforementioned BLUF domain proteins (YcgF, YtvA, and CryB). Unlike canonical bacterial photoreceptors that harbor a sensor domain and variable effector modules (such as kinase, c-di-GMP phosphodiesterase, and adenylate cyclase), the full length (17 kDa) of BlsA that lacks the effector domain is shorter than that of other blue light-sensing proteins (Ppr, BlrP1, and OaPAC; 40 to 70 kDa) (16–19). However, short BLUF domain proteins, such as BlrB, SnfB, and TePixD without additional long effector scaffolds, have also been reported (20, 21).

Interestingly, several interacting partners (such as Fur, AbaR, AcoN, and BfmR) for a short BLUF protein, namely, BlsA, have been identified in A. baumannii strains, indicating that BlsA could modulate diverse cellular responses (such as iron uptake, acetoin/butanediol metabolism, biofilm formation, and quorum sensing) by positively or negatively associating with other proteins in a light-dependent or temperature-dependent manner (7, 15, 22, 23). Direct interactions between BlsA and Fur could lead to the expression of gene clusters encoding the acinetobactin siderophore and could thereby facilitate the survival of A. baumannii ATCC 19606 cells under iron-deprived conditions at 23°C in the dark; however, this effect was not noted under light or at 30°C (22). The production of AbaI-mediated acyl-homoserine lactones (AHLs) was found to be modulated by the AbaR-BlsA complex at 23°C in the dark; however, this effect was abolished under blue light, following which the quorum quenching system was functional in A. baumannii ATCC 17978 (7). Acetoin catabolism governed by the *acoABC* operon products was enhanced at 23°C under light in Acinetobacter strain 5-2Ac02 by blocking the binding of the AcoN repressor to the promoter of the *acoABC* cluster, indicating that BlsA serves as an antirepressor of the acetoin catabolic operon in a BlsA-canonical manner (15). The immunoprecipitation and microscale thermophoresis of BlsA with BfmR, a response regulator of the BfmS-BrmR two-component system, revealed that their tight interaction and conformational changes upon photoactivation were responsible for controlling various virulence-related traits, including biofilm formation (23, 24). In the present study, the putative novel binding partner (BipA) of the photoreceptor BlsA in A. baumannii ATCC 17978 was identified via transcriptome analysis and through diverse databases (protein-protein interaction database, synteny maps, and bacterial genomes). Moreover, their direct interaction was verified using the bacterial adenylate cyclase two-hybrid system (BACTH). Taken together, our findings provide molecular insight into BlsA-mediated, light-dependent phenotypic changes that are linked to membrane integrity and biofilm formation in A. baumannii strains. We also propose the ecological implications of BlsA loss during the evolutionary adaptation of clinical A. baumannii isolates.

## RESULTS

### Synteny analysis of *blsA* and prediction of BlsA-interacting partners.

Whole-genome analyses of 12 Acinetobacter species (a total of 18 strains), including four A. baumannii strains, revealed 18 *blsA* (purple-colored in Fig. S1A) homologs and 6 homologs of the uncharacterized YgiW-like gene (red-colored in Fig. S1A; hereinafter referred to as the *bipA*, BlsA-interacting protein A). Neither *blsA* nor *bipA* was present in the genomes of three strains, including a clinical isolate of multidrug-resistant (MDR) A. baumannii NCCP 16007 (25). Two homologs of the *blsA* gene in *A. oleivorans* DR1 (ALOE_05860 and ALOE_10045) exhibited different upstream genes (*cspG* and β-lactamase, respectively) of *blsA.* This observation led us to investigate the role of BlsA and its neighboring BipA under light conditions through transcriptome, bacterial two-hybrid system, and comprehensive deletion analyses. The same guanine-cytosine (GC) ratio (35%) of conserved *blsA* in all cases and the absence of *bipA* in different genomic backgrounds (GC ratio of approximately 39 to 42%) suggested the gain or loss of light-responsive proteins during evolutionary adaptation (Fig. S1A and B) (26). Neither the *bipA* homolog nor the *blsA* homolog was detected in three clinical isolates (A. baumannii SDF, A. junii SH205, and A. baumannii NCCP 16007). Many clinical isolates of Acinetobacter species, including the *blsA*-deficient strains, appeared to have multiple transposases (ranging from 9 to 22 in the three aforementioned A. baumannii strains) and insertion sequence (IS) elements (ranging from 5 to 19), which might be attributable to the rearrangement of their genomes and the resultant loss of *bipA* and *blsA* (25, 27, 28). The loss of the *bipA* and *blsA* light-sensing capability could be one of the nosocomial pathogens’ *in vivo* adaptive strategies (29, 30). It was partly supported by the results of our simple BlsA-dependent growth tests of the wild-type (ATCC 17978) strain and the *blsA*-deficient strain (NCCP 16007) on agar plates under light conditions (*P* < 0.001) (Fig. S1C; Table S1). Deletion and complementation assays using the ATCC 17978 and NCCP 16007 strains demonstrated the requirement of BlsA or BipA for their survival under various light intensities.

10.1128/msystems.00897-22.1 FIG S1 Synteny analysis of *blsA* and the protective effect of *blsA* from blue light. (A) Presence and absence of *blsA* and *bipA* in representative members of the genus Acinetobacter. MaGe software was used in Fig. S1A. The same colors indicate the conservation of genomic content for each gene. The homolog of *blsA* was not detected in 3 genomes among a total of 18 analyzed strains (2 copies of the *blsA* gene are present in *A. oleivorans*) DR1, A. nosocomialis RUH2624, and A. baylyi ACIAD2125. 7 *blsA* homologs in our tested 6 strains (*A. oleivorans* DR1 AOLE_05860, *A. oleivorans* DR1 AOLE_10045, A. nosocomialis RUH2624 ACIRUv1_270092, A. nosocomialis RUH2624 ACIRUv1_920004, A. baylyi ACIAD1499, and A. baylyi ACIAD2125) exhibit nonconserved synteny regions next to the *blsA* gene (the neighborhood genes are colored in gray, and the antibiotic resistance genes are colored in glaucous). Coding sequences (CDSs) are aligned with corresponding CDSs in the ATCC 17978 strain. The GC contents (%) of the sequenced chromosome are shown (in red). (B) Phylogenetic relationships of BLUF proteins in the genus Acinetobacter. (C) Light susceptibility tests of the ATCC 17978 and NCCP 16007 strains at 23°C. Blue light susceptibility tests were performed on an LB agar plate after inoculating the cells (10^7^ CFU/mL) under blue light (0 to 16 W/m^2^). The cell survival rate was calculated as the relative percentage in comparison with the number of colonies counted under dark conditions. Download FIG S1, PDF file, 1.0 MB. Copyright © 2023 Yang et al. 2023 Yang et al. https://creativecommons.org/licenses/by/4.0/ This content is distributed under the terms of the Creative Commons Attribution 4.0 International license.

10.1128/msystems.00897-22.3 TABLE S1 Light susceptibilities of clinical A. baumannii isolates. Blue light (intensity 0 to 16 W/m^2^) susceptibilities were determined on a Luria-Bertani (LB) agar plate inoculated with each cell culture (10^7^ CFU/mL). Cell survival rates were calculated as the relative percentages in comparison with the numbers of colonies counted at 0 W/m^2^. Sublethal intensities of blue light were defined as intensities that killed 25% (BL25) and 50% (BL50) of the total surviving bacteria. *: P, presence; A, absence. **: SU, Seoul; GW, Gangwon-do; BS, Busan; GJ, Gwangju; GN, Gyeongsangnam-do; CB, Chungcheongbuk-do; JN, Jeollanam-do; JB, Jeollabuk-do; DG, Daegu; GG, Gyeonggi-do; IC, Incheon; GB, Gyeongsangbuk-do; NA, not available. Download Table S1, DOCX file, 0.02 MB. Copyright © 2023 Yang et al. 2023 Yang et al. https://creativecommons.org/licenses/by/4.0/ This content is distributed under the terms of the Creative Commons Attribution 4.0 International license.

**FIG 1:**
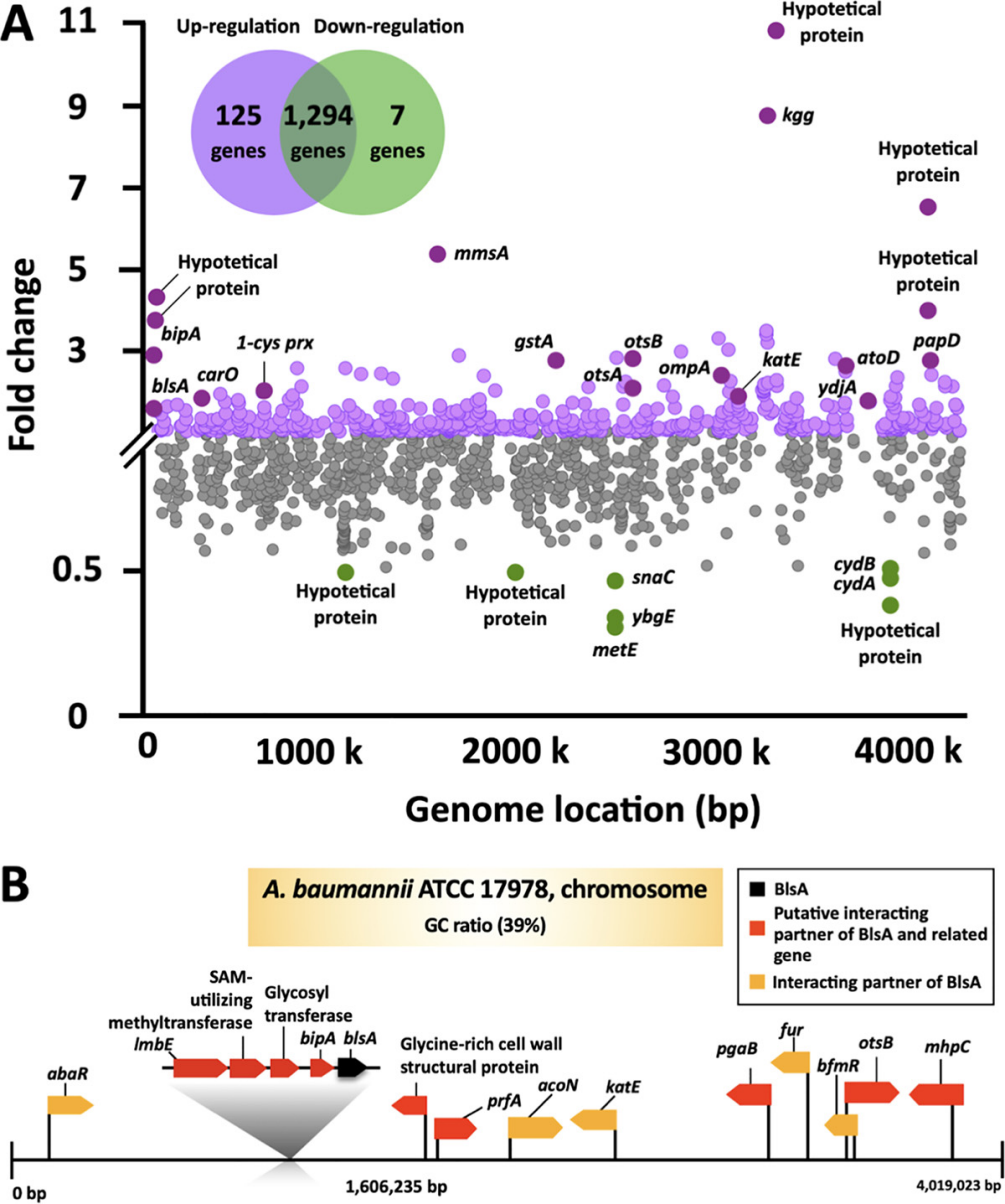
Prediction of BlsA-interacting partners, based on transcriptome and database analyses. (A) Transcriptome analyses of ATCC 17978 cells under blue light or dark conditions. The cells were incubated for 12 h under blue light (4 W/m^2^) or under dark conditions in LB media (10 mL) at 23°C. (B) Nomination of putative binding partners, based on the STRING protein-protein interaction database. A total of 9 genes as novel BlsA-interacting partners (red arrows), along with five previously reported BlsA-interacting proteins (yellow arrows), have been depicted in the chromosome of the ATCC 17978 strain (contig size, 4,019,023 bp; GC ratio, 39%).

The potential BlsA-interacting partners and light-responsive proteins were nominated based on our light-dependent (blue light intensity of 4 W/m^2^ at 23°C) transcriptome analyses and the Search Tool for the Retrieval of Interacting Genes/Proteins (STRING) interaction database. Several stress defense genes (such as *katE*, *1-cys prx*, *otsA*, and *gstA*) were upregulated along with *blsA*, indicating a cellular strategy in response to photooxidative stress. Moreover, the modulation of a large set of genes (125 upregulated [>1.5-fold upregulation, purple colored] and 7 downregulated [<0.5-fold downregulation, green colored]) was monitored during light exposure (see Data Set S1 for the complete list of transcriptomes). The BlsA-dependent regulation of *katE* was also genetically demonstrated in a previous study, despite no direct biophysical evidence (31). Interestingly, many genes encoding outer membrane porins, such as OmpA, CarO, Omp33-36, PxpA, and AqpZ, were also induced during light conditions, indicating the light-dependent alteration of permeability and antibiotic resistance (Fig. 1A; Table S2) (32). The light induction of both *bipA* (2.87-fold) and *blsA* (1.51-fold) as well as their close genetic location prompted us to propose their functional linkage for cellular survival under light conditions. A total of 9 newly predicted proteins (red-colored in Fig. 1B) and 5 previously reported BlsA-interacting proteins (AbaR, AcoN, KatE, Fur, and BfmR) as putative BlsA-interacting partners are depicted in Fig. 1B (orange-colored) (7, 15, 22, 23, 31). The STRING database contains data derived from an automated computational analysis that used the following gene prediction methods: neighborhood on chromosome, phylogenetic co-occurrence, and gene coexpression studies (33). A required confidence score (RC) of >0.4 was selected as the threshold for the prediction of protein-protein interactions. Using the parameter of gene neighborhood, four proteins (BipA, LmbE, the putative glycosyl transferase, and SAM-utilizing methyltransferase) were selected as direct BlsA-interactors (RC: approximately 0.455 to 0.498), and a putative glycine-rich cell wall structural protein was nominated as an indirect partner of BlsA (RC: 0.446). Three proteins (PrfA, PgaB, and KatE) were also nominated as indirect partners of BlsA by using the gene coexpression parameter with an RC range of 0.452 to 0.493. Moreover, the putative indirect partners of BlsA derived via the gene cooccurrence method were OtsB and MhpC, with RC values of 0.492 and 0.474, respectively. Among these STRING-based nominations, three genes (*bipA*, *katE*, and *otsB*) were upregulated by blue light in our transcriptome analysis (Fig. 1A and B). However, because the KatE and OtsB were predicted to be the indirect partners of BlsA, we attempted to analyze the direct interactions of BlsA with BipA, considering their proximity and bioinformatic clues.

10.1128/msystems.00897-22.4 TABLE S2 List of genes that were upregulated or downregulated in the A. baumannii ATCC 17978 cells under blue light irradiation. Total RNA was extracted from exponentially grown (OD_600_ of approximately 0.5) ATCC 17978 cells under blue light treatment (4 W/m^2^) at 23°C. Relative transcript abundances are presented as fragments per kilobase of exon per million mapped sequence reads (FPKM). Genes exhibiting fold changes (FPKM values of blue light-treated cells/FPKM values of control cells) of greater than 1.5 and less than 0.5 were regarded as upregulated and downregulated genes, respectively. Genes with FPKM values below 50 in both the dark control and the blue light-irradiated samples were discarded. Download Table S2, DOCX file, 0.02 MB. Copyright © 2023 Yang et al. 2023 Yang et al. https://creativecommons.org/licenses/by/4.0/ This content is distributed under the terms of the Creative Commons Attribution 4.0 International license.

10.1128/msystems.00897-22.10 DATA SET S1 Complete list of transcriptomes of the A. baumannii ATCC 17978 cells under blue light irradiation. Download Data Set S1, XLSX file, 0.4 MB. Copyright © 2023 Yang et al. 2023 Yang et al. https://creativecommons.org/licenses/by/4.0/ This content is distributed under the terms of the Creative Commons Attribution 4.0 International license.

### Direct *in vivo* interactions of BlsA with BipA.

**FIG 2:**
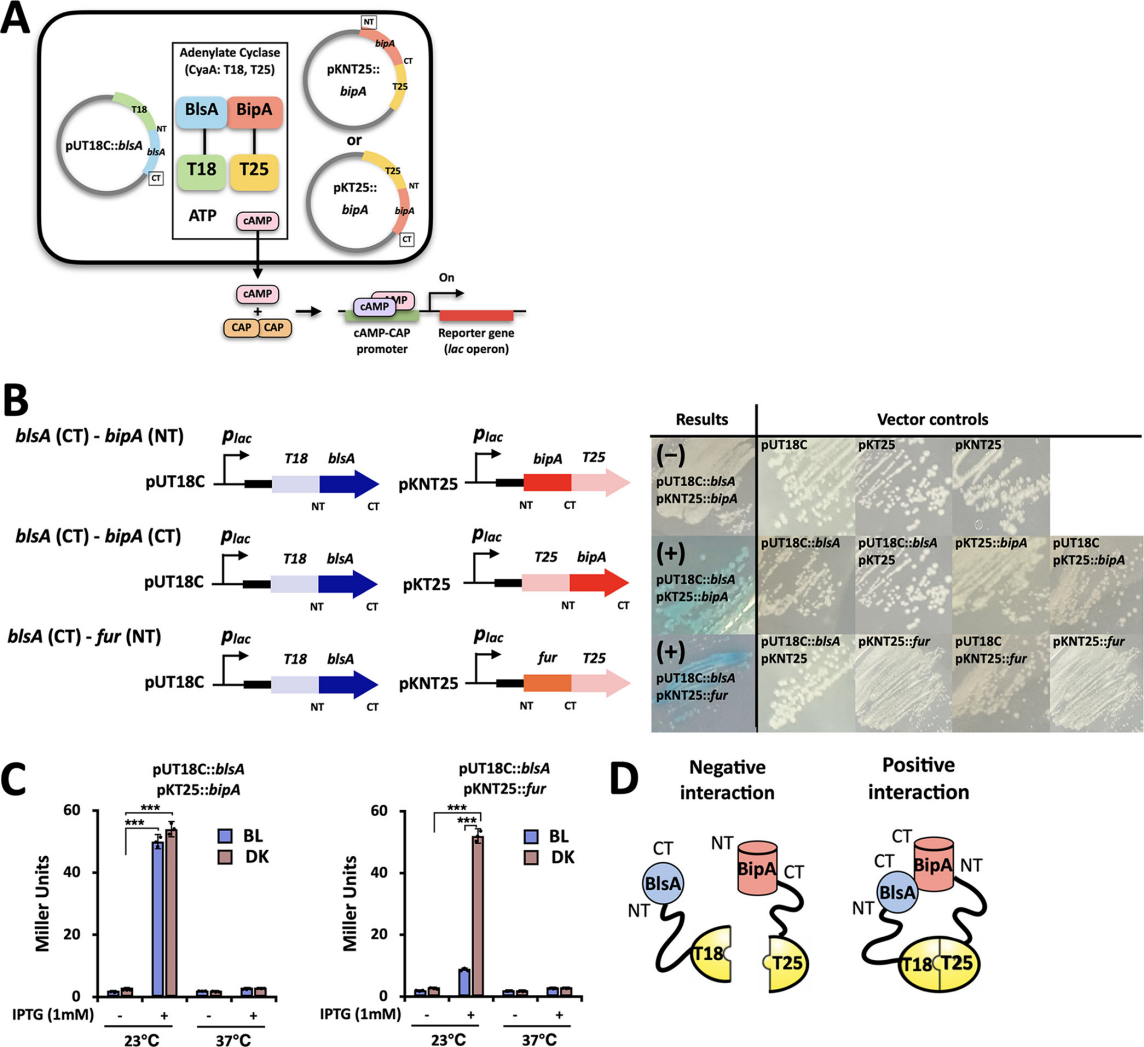
Protein interactions with BlsA, as determined by the BACTH system in the *cyaA*-deleted strain E. coli BTH101. (A) Schematic overview of the BACTH system, based on the functional complementation of B. pertussis CyaA subunits (T18 and T25). (B) *In vivo* interactions of BlsA, BipA, and Fur. Constructed plasmids for the fused proteins (left). The corresponding trial results in petri dishes were visualized via IPTG-mediated induction, either as positive (+) interactions (blue-colored colonies) or as negative (−) interactions (white-colored colonies). (C) Determination of interactions based on β-galactosidase activity. Fivefold higher galactosidase activity (expressed in Miller units) than the negative control was considered to be a positive interaction. All of the tested samples were incubated under blue light (4 W/m^2^) or dark conditions at 23°C for 24 h (N = 3 biological replicates). Statistical analyses were performed using Student’s *t* test (two-tailed) for comparisons of two groups (***, *P* < 0.001). (D) Putative negative/positive interaction models of the BACTH system. BL, blue light irradiation; DK, dark control.

The BACTH system was used to investigate the *in vivo* interactions of BlsA with the possible candidate BipA. The Fur protein of A. baumannii was used as a positive control (22, 33). The protein structures and interacting surfaces of BlsA and its physical association with other proteins were predicted using several bioinformatic tools (such as Phyre^2^, GalaxyWEB software, and CLC Genomics Workbench) in order to clone the critical interacting parts of all of the tested proteins into the pUC19 and pSU40 vectors of the BACTH system (Fig. S2). Our bioinformatic findings related to the homology modeling, local amino acid complexity, and hydrophobicity revealed the possible folding structures and potential binding sites of putative partners with BlsA. BlsA has a canonical BLUF domain and is predicted to have a C-terminal interacting region. The other target protein, namely, BipA, had C-terminal putative BlsA-binding sites with N-terminal β-barrel-like transmembrane regions (Fig. S2). A complete set of six vectors containing full target proteins that were physically connected to a part of adenylate cyclase were generated to monitor their IPTG-controlled interactions under light conditions (Fig. 2A). Positive interactions could be visualized via the IPTG-mediated induction of fused protein expression only when the proposed C-terminal regions of target proteins were placed in our constructed pKT25 vector system (Fig. 2B–D). Interestingly, the BlsA-BipA interaction was maintained regardless of light conditions, and a positive BlsA-Fur interaction was also verified under dark conditions *in vivo* (Fig. 2B and C). In addition, the β-galactosidase activity of the tested strains harboring the complemented adenylate cyclase demonstrated the occurrence and degree of positive interactions under varied light and low temperature (23°C) conditions, suggesting that protein folding or interactions could also be affected by light and temperature (Fig. 2C and D) (34, 35).

10.1128/msystems.00897-22.2 FIG S2 Homology modeling of BlsA and BipA. (A) Homology modeling of A. baumannii BlsA (PDB entry 6W6Z, 100% sequence identity) and BipA (PDB entry 1NNX, 42% sequence identity). Each protein model was spectrum colored from the N terminus (NT, blue) to the C terminus (CT, red). The flavin chromophore (FMN and FAD) is shown in a ball-and-stick representation with gray carbon atoms, blue nitrogen atoms, red oxygen atoms, and an orange phosphate atom in the green structure of BlsA or as blue colored in the gray structure. The 3D models were initially constructed using Phyre^2^ and were refined without ligands using GalaxyWEB software. Each possible ligand was fitted in the ordered electron density using 3DLigandSite. GAL, β-galactopyranose; GLC, ɑ-d-glucopyranose; BGC, β-d-glucose; LAT, large-neutral amino acid transporter. (B) Local sequence complexity analyses of BlsA and BipA. The y axis shows the K2 measure of the local sequence complexity. (C) Hydrophobicity (transmembrane region of >1.8) of the proteins (see Materials and Methods, synteny analysis of *blsA* and homology modeling of BlsA). All final products were visualized and assessed using CLC Genomics Workbench v.10.0.1 (Qiagen, Germany). Download FIG S2, PDF file, 0.8 MB. Copyright © 2023 Yang et al. 2023 Yang et al. https://creativecommons.org/licenses/by/4.0/ This content is distributed under the terms of the Creative Commons Attribution 4.0 International license.

### Light-dependent expression of *blsA* and its partners.

**FIG 3:**
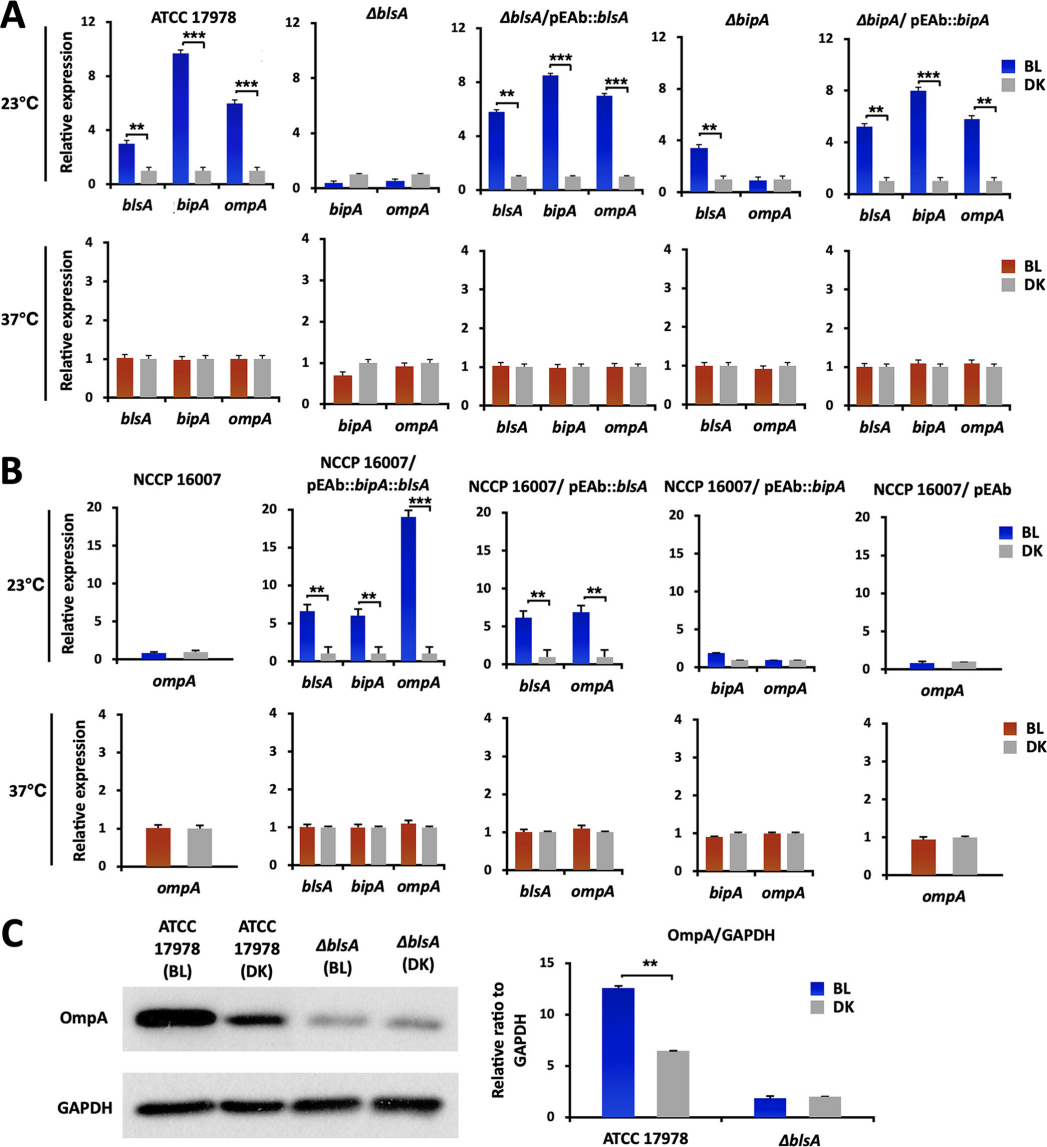
Expression levels of *blsA*, *bipA*, and *ompA* in the ATCC 17978 and NCCP 16007 strains. (A) Relative expression levels of *blsA*, *bipA*, and *ompA* in the ATCC 17978 strains under blue light (4 W/m^2^) or dark conditions at 23°C. (B) The expression of *ompA* and the transformed genes (*blsA* and *bipA*) in the NCCP 16007 strains under the same conditions as those for the ATCC 17978 strains. All of the tested samples were harvested for mRNA extraction in the mid-exponential phase (OD_600_ of approximately 0.5) after 12 h of incubation in LB media (10 mL). The expression levels of the target genes were normalized according to the expression level of 16S rDNA. (C) OmpA-expression of ATCC 17978 and the *blsA* mutant in response to blue light. The broad-range anti-GAPDH was used as a loading control. Statistical analyses were performed using Student’s *t* test (two-tailed) for comparisons of two groups (**, *P* < 0.01; ***, *P* < 0.001). BL, blue light irradiation; DK, dark control.

Our transcriptome and qRT-PCR analyses demonstrated that the expression of *blsA* and its partner *bipA* was induced by light and temperature in A. baumannii ATCC 17978 (Fig. 3A and B). Because of the light-dependent upregulation of the *ompA* gene in our transcriptome analysis, it was worth investigating the contribution of light-dependent BlsA to the expression of *ompA*, which could alter outer membrane integrity and antibiotic resistance (36). Interestingly, the light-dependent expression of *bipA* and *ompA* was eliminated in the *blsA* mutant; however, the light-induced expression of both of these genes was recovered in our complementation assay, suggesting that the BlsA-mediated direct or indirect modulation of target genes occurs through light-sensing mechanisms (Fig. 3A). High temperature (37°C) abolished the photoupregulation of both the *bipA* and *ompA* genes, which was also supported by the results of the previous study, which showed that the expression of *blsA* could be induced by low temperature and light in A. baumannii ATCC 17978 (Fig. 3A and B) (37). However, the direct DNA-binding regulator controlling these systems has not yet been identified. Interestingly, the deletion of *bipA* diminished the light-dependent induction of *ompA.* However, in the complemented strain, *ompA* expression was restored under light conditions (Fig. 3A). Both *blsA* and *bipA* were individually or together transformed into A. baumannii NCCP 16007 that lacked both of the corresponding genes in order to monitor their expression dependence (Fig. 3B). Surprisingly, light dependence of the expression of three tested genes (*blsA*, *bipA*, and *ompA*) could be observed in the ectopic *blsA*-expressing NCCP 16007 strain at 23°C but not at 37°C. No effect of light was observed on the expression of *ompA* in the NCCP 16007 strain. However, ectopically expressed BlsA and not BipA caused the upregulation (6.89-fold) of *ompA*, with profoundly higher expression levels (19.01-fold) being noted when both of the genes were ectopically expressed. The higher BlsA-mediated induction (2-fold) of the OmpA protein under blue light in the ATCC 17978 was also confirmed via Western blot assay (Fig. 3C). Interestingly, the expression levels of OmpA in the *blsA* mutant were relatively low under blue light and dark conditions (Fig. 3C). These results indicated that BlsA, with the help of BipA, may lead to the upregulation of OmpA by interacting with uncharacterized direct DNA-binding regulators.

### BlsA-dependent modulation of membrane integrity and meropenem uptake.

**FIG 4:**
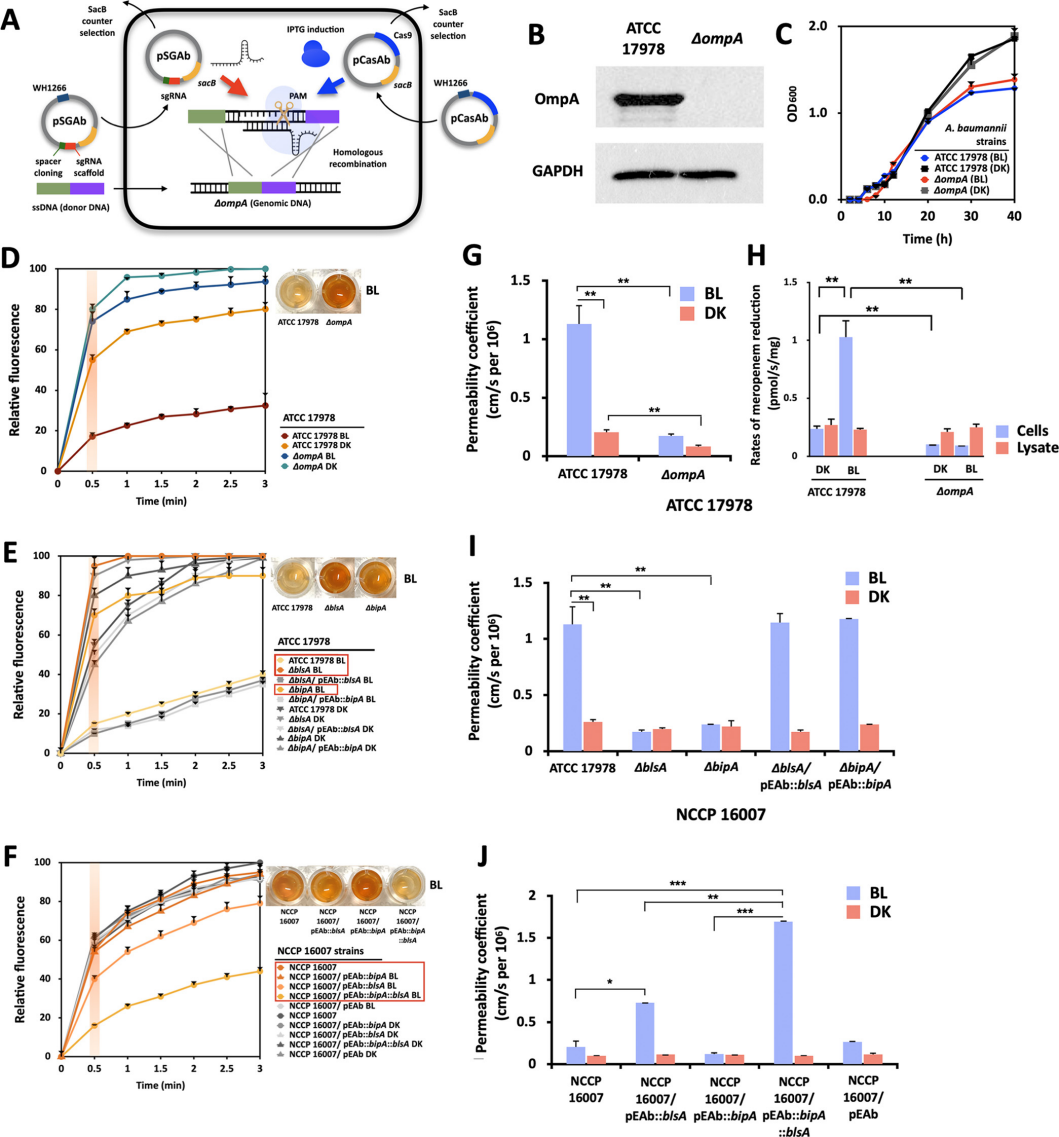
Light-mediated modulation of membrane integrity and meropenem uptake. (A) Schematic overview of CRISPR/Cas9-based gene knockout of *ompA.* (B) Deletion of OmpA, confirmed via Western blotting. (C) Growth curves of ATCC 17978 and the *ompA* mutant under light and dark conditions. (D) EtBr permeation of the *ompA*-knockout mutant, (E) *blsA*- and *bipA*-deleted mutants, and corresponding gene-complemented ATCC 17978 strains. (F) EtBr uptake of the *blsA*- and *bipA*-transformed NCCP 16007 strains. The intensity of fluorescence emission was detected at excitation and emission wavelengths of 515 and 590 nm, respectively. (G) The permeability coefficient of meropenem and (H) the meropenem reduction rates in the ATCC 17978 strain and its *ompA*-deleted mutant strain. (I) The permeability coefficient of the *blsA-* and *bipA*-knockout mutants in the ATCC 17978 strain and (J) the NCCP 16007 strain. The diffusion rates of meropenem through the outer membrane were determined by measuring the rates of meropenem reduction in bacterial cells and lysates under blue light (4 W/m^2^) or dark conditions at 23°C (N = 3 biological replicates). Statistical analyses were performed using Student’s *t* test (two-tailed) for comparisons of two groups (*, *P* < 0.05, **; *P* < 0.01; ***, *P* < 0.001). BL, blue light irradiation; DK, dark control.

The deletion of *ompA* from A. baumannii ATCC 17978 was performed using the CRISPR/Cas9 system to monitor the light-mediated changes and the involvement of OmpA in membrane integrity with the presence and absence of OmpA (Fig. 4A). Nonproduction of OmpA in the mutant was checked via Western blotting, and no growth defect of the mutant was observed (Fig. 4B and C). Since OmpA is the most abundant major outer membrane porin (e.g., approximately 100,000 copies/cell in E. coli), we speculated that the increased light-dependent expression levels of OmpA in the outer membrane would reduce the membrane permeability of the small hydrophobic lipophilic dye EtBr (32, 36, 38). Stronger EtBr fluorescence was observed under dark conditions for 3 min; however, this effect was not observed under light conditions. This tendency decreased when *ompA* was deleted from the ATCC 17978 strain, suggesting that large amounts of light-induced OmpA could decrease EtBr permeation (Fig. 4D). When the modulators of *ompA* expression (BlsA and BipA) were deleted from A. baumannii ATCC 17978, more fluorescence was detected, indicating that both of these genes are required for the light-dependent reduction of the membrane permeation of EtBr (Fig. 4E). Our complementation assay recovered the tendency of light-dependent membrane permeation of EtBr to the same degree as that noted in the wild-type strain. This finding was also supported by the fact that ectopically expressed *blsA* or *blsA* together with *bipA* could lead to the same results in the NCCP 16007 strain (Fig. 4F). However, consistent with findings in the wild-type strain, the ectopic expression of *bipA* alone was not sufficient for changing light-dependent EtBr permeation in the NCCP 16007 strain. An empty vector was used as a negative control in all of the ectopic expression experiments.

Since OmpA contributes to the transport of hydrophilic and small β-lactam antibiotics into cells, OmpA-mediated antibiotic transport using meropenem, a modern β-lactam antibiotic, was tested under light conditions (36–39). Light-dependent meropenem penetration was assessed by monitoring the rate of decrease in fluorescence (wavelength: 297 nm) during meropenem uptake for 10 min. The prepared intact cell sample was used for the meropenem uptake assay, and other cell cultures were sonicated for use as negative controls in assessing the β-lactamase-mediated degradation and cellular binding of meropenem (see Materials and Methods). The reduction of the meropenem concentration in the sample was measured, and the corresponding permeability coefficients were calculated. The results revealed that the *ompA* mutant exhibited a lower meropenem influx than did the ATCC 17978 strain, indicating the effect of OmpA on meropenem permeation under both blue light and dark conditions (Fig. 4G). Moreover, the meropenem influx increased in ATCC 17978 cells under blue light, probably because the BlsA-dependent light induction of OmpA resulted in higher meropenem uptake into cells in the presence of OmpA (Fig. 4G) (39, 40). The rates of meropenem reduction were also quantified using cell lysates of the wild-type and *ompA* mutant strains. The results revealed that the amounts of β-lactamase and cellular components could not contribute to an increase in the permeability coefficient in the wild-type strain under light conditions (Fig. 4H; Table S3). A light-dependent increase in meropenem permeability (1.1 cm/s per 10^6^ cells) could not be detected in either of the *blsA* or *bipA* mutants (0.20 to 0.25 cm/s per 10^6^ cells); however, the light-dependent effect was recovered when each gene was individually complemented in both of the mutants (Fig. 4I; Table S3). Interestingly, the same light-dependent meropenem permeability was negligible in the NCCP 16007 strain. While the addition of *blsA* or *blsA* together with *bipA* boosted meropenem permeability, the addition of *bipA* alone did not (Fig. 4J; Table S3).

10.1128/msystems.00897-22.5 TABLE S3 Determination of meropenem uptake rates. The uptake rate of meropenem into cells was estimated by measuring the concentration of meropenem in the supernatant. The cells were grown at 23°C for 12 h in LB media (50 mL) with aeration by shaking under blue light (4 W/m^2^) or dark conditions. The cells were washed once with potassium phosphate buffer (pH 7.0, 50 mM) containing MgCl_2_ (5 mM). The washed cells were resuspended in the same buffer (OD_600_ of approximately 0.8). See Text S1 (Determination of EtBr and meropenem influx) for more detailed information. *, Rates of meropenem reduction (pmol/s/mg). **, Permeability coefficient (see Text S1, determination of EtBr and meropenem influx). BL, blue light irradiation; DK, dark control. Download Table S3, DOCX file, 0.02 MB. Copyright © 2023 Yang et al. 2023 Yang et al. https://creativecommons.org/licenses/by/4.0/ This content is distributed under the terms of the Creative Commons Attribution 4.0 International license.

### BlsA-dependent changes in light susceptibility and biofilm formation.

**FIG 5:**
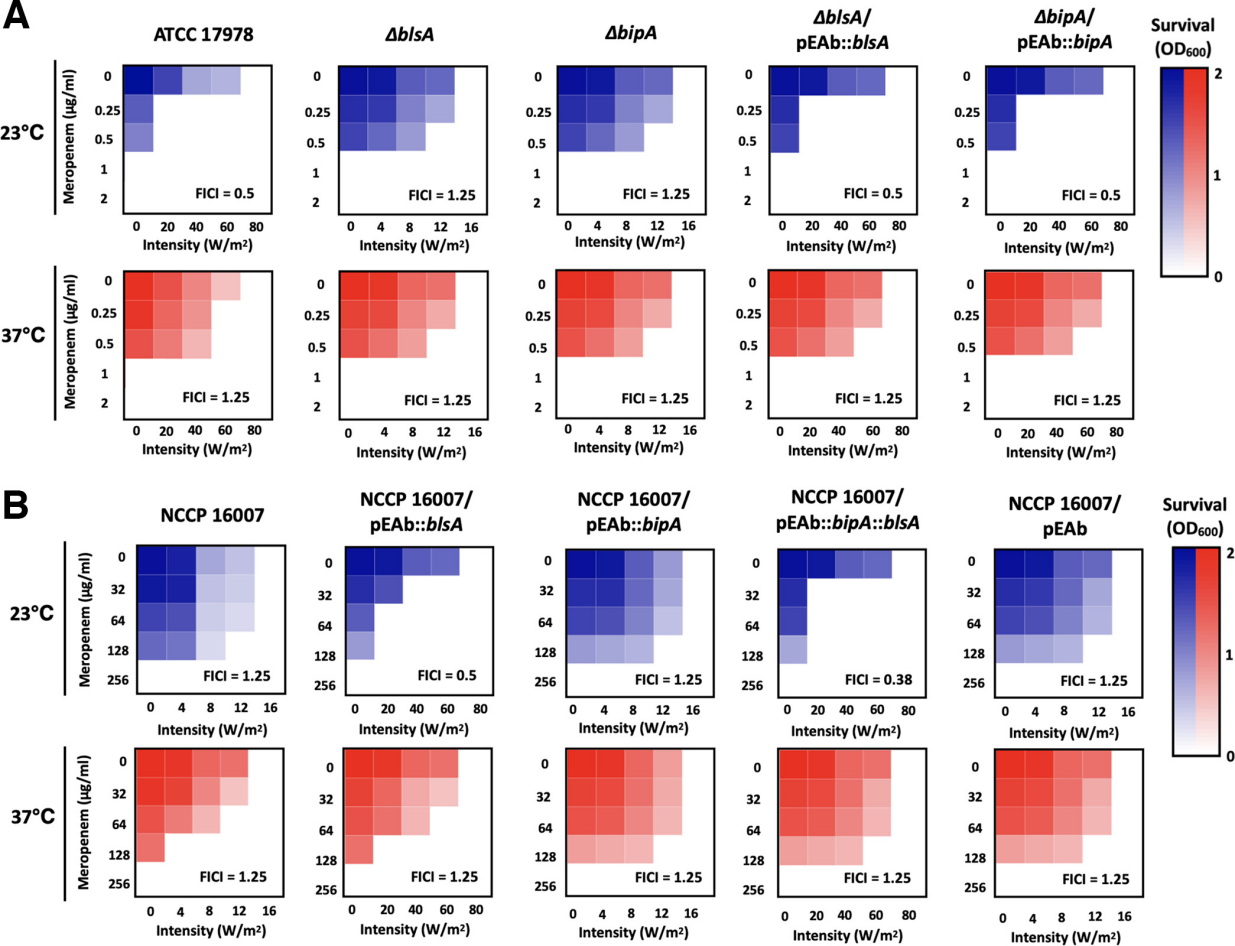
Synergistic activity between blue light and meropenem. (A) Checkerboard assay of the ATCC 17978 strain, its *blsA-* and *bipA*-knockout mutants, and the gene-complemented strains. (B) Checkerboard assay of the NCCP 16007 strain and its *blsA*- and *bipA*-transformed strains. The density of each cell culture was determined at 23°C or 37°C under blue light (0 to 80 W/m^2^) or dark conditions. The data represent the mean OD_600_ values of three biological replicates. Dark blue/red regions represent higher cell densities. Synergy is defined as a fractional inhibitory concentration index (FICI) of ≤0.5. BL, blue light irradiation; DK, dark control.

To assess the susceptibility of the ATCC 17978 strain (possessing *blsA* and *bipA*) and the NCCP 16007 strain (lacking *blsA* and *bipA*) to meropenem with/without light conditions, the fractional inhibitory concentration index (FICI) of meropenem was measured under blue light at 23°C or 37°C (Fig. 5; Table S4). Decreased meropenem susceptibility was observed under both blue light and dark conditions in the *ompA* mutant, suggesting that OmpA could act as the main channel for transporting meropenem across the membrane (Table S4). The deletion of BlsA or BipA increased the light-mediated mortality rates (80 W/m^2^ versus 16 W/m^2^ in liquid culture) of ATCC 17978 cells under meropenem treatment. However, no significant changes in such tendencies were noted at a higher temperature (37°C). Moreover, complementation at 23°C recovered these light-dependent bactericidal events. These results suggest that both BlsA and BipA could make cells more susceptible to light under meropenem or vice versa (Fig. 4I and 5A). Interestingly, consistent with this speculation, the light susceptibility of the NCCP 16007 strain intrinsically lacking BlsA and BipA was found to differ with the ectopic expression of BlsA in response to light or a combination of light and meropenem (Fig. 5B). Moreover, a synergistic bactericidal effect (<0.5 FICI) of meropenem under light conditions was noted in the ATCC 17978 cells at 23°C but not at 37°C. The same synergistic effect was noted in the ectopic *blsA*-expressing NCCP 16007 cells, and the ectopic expression of both *blsA* and *bipA* attributed to a stronger synergistic bactericidal effect (<0.38 FICI) (Fig. 5B).

10.1128/msystems.00897-22.6 TABLE S4 Synergistic activity of meropenem with blue light against A. baumannii strains. Each well of a 96-well plate was filled with LB broth (200 μL). Following this, the antibiotics and compounds were twofold diluted along the abscissa, and the intensity of the blue light was increased along the ordinate (from 0 to 80 W/m^2^). After incubating at 23°C or 37°C with bacterial suspensions (10^6^ CFU/well), the absorbance of each well at 600 nm was measured. The FIC was calculated as the MIC/lethal intensity when the antibiotics and blue light were used in combination, divided by the MIC/lethal intensity when used alone. The FICI is defined as the sum of the FICs of two compounds, and synergy is defined as a FIC index of ≤0.5. BL intensity^a^/BL intensity^b^, MIC of blue light intensity (W/m^2^) in the presence/absence of meropenem. MPN^a^/MPN^b^, MIC of meropenem (μg/mL) in the presence/absence of blue light. FICI, FIC index. *, *P* < 0.05 (BL intensity^a^/BL intensity^b^, N = 3 biological replicates). ^#^, *P* < 0.05 (MPN^a^ versus MPN^b^, N = 3 biological replicates). Download Table S4, DOCX file, 0.02 MB. Copyright © 2023 Yang et al. 2023 Yang et al. https://creativecommons.org/licenses/by/4.0/ This content is distributed under the terms of the Creative Commons Attribution 4.0 International license.

**FIG 6:**
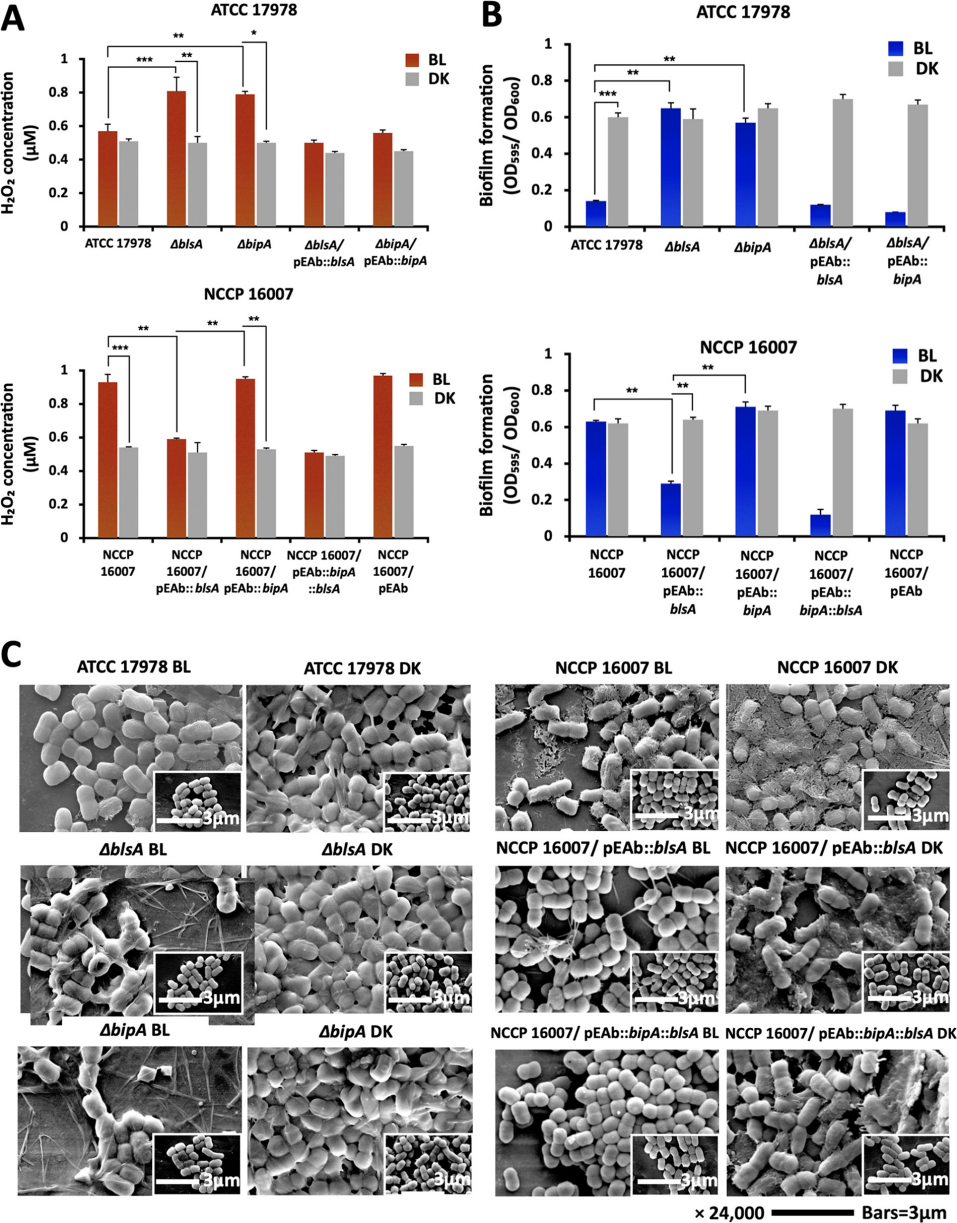
Light-induced stress response of A. baumannii strains. (A) H_2_O_2_ production and (B) biofilm formation ability of the ATCC 17978 strain, its *blsA*- and *bipA*-knockout mutants, the gene-complemented strains, and the NCCP 16007 strain (N = 3 biological replicates). Statistical analyses were performed using Student’s *t* test (two-tailed) for comparisons of two groups (*, *P* < 0.05; **, *P* < 0.01; ***, *P* < 0.001). (C) Scanning electron microscopic (SEM) images of the ATCC 17978 strain and its *blsA*- and *bipA*-knockout mutants, as well as the NCCP 16007 strain and its *blsA*- and *bipA*-transformed mutants. The mid-exponentially grown cells (OD_600_ of approximately 0.5) were used to prepare for the SEM observations. All of the tested samples were incubated at 23°C under blue light (4 W/m^2^) or dark conditions. BL, blue light irradiation; DK, dark control.

Because light can impose oxidative stress on cells, we sought to measure the generation of hydrogen peroxide (H_2_O_2_) in the supernatants of ATCC 17978 and NCCP 16007 strain-grown cultures under blue light (41, 42). BlsA-dependent changes in H_2_O_2_ production were observed in both ATCC 17978 and ectopic *blsA*-expressing NCCP 16007 strains, suggesting that BlsA-mediated light responses and the consequent changes in membrane integrity could protect cells from light-induced oxidative stress (Fig. 6A). All of the complementation assays and comparisons of the two wild-type A. baumannii strains supported our finding of the BlsA- or BipA-mediated reduction of H_2_O_2_ production. Moreover, the presence or absence of BlsA or BipA affected biofilm formation in the ATCC 17978 cells under light in the stationary phase at 23°C; however, no light-dependent change in biofilm formation was noted in the NCCP 16007 strain (Fig. 6B). The ectopic *blsA*-expressing cells had the same tendency of biofilm formation as did the ATCC 17978 strain (Fig. 6B). The deletion of *blsA* or *bipA* individually from the ATCC 17978 strain increased the surface appendages under light (Fig. 6C). Unlike the ATCC 17978 cells, the NCCP 16007 strain formed thick and condensed biofilms with surface appendages; however, the ectopic addition of BlsA caused the loss of surface appendages and produced smooth cellular surfaces, possibly reducing biofilm formation under light conditions (Fig. 6C). Our data indicated that BlsA and BipA together were involved in the changes of all of the tested characteristics (H_2_O_2_ production, biofilm formation, and production of surface appendages) under light conditions. However, the underlying molecular mechanisms of BlsA for all of the tested characteristics remain to be investigated.

## DISCUSSION

The light-dependent modulation of bacterial physiology and behaviors is widely present, even in human pathogens, such as A. baumannii, Staphylococcus aureus, Listeria monocytogenes, and Pseudomonas aeruginosa; however, the roles of these light-sensing proteins in bacterial survival, adaptation, and virulence remain unclear (43). The photoreceptor RsbL of L. monocytogenes, BphP of P. aeruginosa, and BlrP1 of K. pneumoniae could respond to blue light for ROS defense, virulence factor production, and biofilm formation, respectively; however, their underlying mechanisms and interacting partners remain unknown (19, 44–46). The presence and physiological roles of BlsA containing the BLUF domain only in certain groups of Acinetobacter species are questionable, suggesting that BlsA-mediated, light-responsive bacterial behaviors could be driven by the gain or loss of the *blsA* gene for bacterial long-term survival under a given environment. Notably, our genome comparison revealed that three clinical isolates (A. baumannii SDF, A. junii SH205, and A. baumannii NCCP 16007) lack the *bipA* and *blsA* genes as well as many neighboring genes (Fig. S1A). The NCCP 16007 strain has been reported to be resistant to 14 antibiotics of different classes (e.g., meropenem, polymyxin B, and rifampicin) and possesses 13 antibiotic resistance genes acquired from other pathogens, including K. pneumoniae and P. aeruginosa (25). Similarly, A. junii is also regarded as an MDR strain, with its acquisition of many mobile genetic elements, such as plasmids and IS elements (28, 46, 47). The absence of BlsA in A. baumannii NCCP 16007 resulted in the extreme susceptibility of the NCCP 16007 strain, but not of the BlsA-possessing ATCC 17978 strain, to a relatively low light intensity (16 W/m^2^) within 24 h of incubation on agar plates (Fig. S1A and C). These light-sensitivity data suggested that the maintenance of BlsA with BipA on the genomes may be required to cope with light-mediated stressful environmental conditions but not inside the host. The lack of blue illumination in human internal tissues and the warm body temperature led us to speculate that photoreceptors might not have been necessary for a long period of systemic infection, which may be attributed to the loss of BlsA and/or BipA in several A. baumannii strains (37, 46, 47). However, both proteins may be required for survival and for the pathogenesis of surface-exposed wound/skin infections under light and low-temperature conditions (48).

BipA next to BlsA, a YgiW family protein, belongs to the oligosaccharide oligonucleotide-binding fold (OB fold) domain and may bind to proteins and several small molecules. Thus, the BlsA-BipA interactions may be more complex than expected. However, our tested assays showed the possible direct interaction of BlsA and BipA (Fig. 2B and C) and their linkages to facilitate ROS defense, biofilm formation, and membrane integrity (Fig. 4E, 4I, and 6). Further *in vitro* experiments are required to elucidate their interactions and consequent functions in more detail. PhoP-controlled YdeI, an OB fold family protein from Salmonella enterica that has 28% amino acid identity to BipA, is also known to interact directly with OmpD and to affect the expression of many PhoP-regulated genes. This explains its essential roles in multiple processes (antimicrobial peptide resistance, glucose starvation, and virulence) (49). Similarly, we reported the BipA-mediated modulation of *ompA* expression and the involvement of BlsA with BipA in many tested assays (Fig. 3, 4E, 4I, 5, and 6), suggesting that BipA plays roles in multiple stress responses. OmpA plays important roles in biofilm formation, the maintenance of membrane integrity, and antibiotic transport in many bacterial species, but the regulatory systems of *ompA* and OmpA in Acinetobacter species remain unclear (38, 50). In the present study, the BlsA-dependent induction of *ompA* occurred under light conditions in the ATCC 17978 strain; however, no effect of light on *ompA* was noted in the NCCP 16007 strain. This result suggests that further genetic and biochemical analyses of the regulation of *ompA* are warranted in Acinetobacter species (Fig. 3).

Collectively, our tested BACTH system exhibited direct BlsA-BipA interaction (Fig. 2B and C), and BlsA also appeared to affect the expressions of the *bipA* and *ompA* genes under light conditions. The present study is the first to reveal the light-dependent interaction of BlsA with BipA at low temperatures in pathogenic A. baumannii, which could result in changes in many characteristics, including meropenem resistance and biofilm formation. The gain or loss of BlsA together with BipA through their evolutionary processes may be beneficial for the survival of Acinetobacter species in response to light.

## MATERIALS AND METHODS

### Bacterial strains, culture conditions, and determination of blue light susceptibility.

The bacterial strains used in the present study are listed in Table S5. The genomes of the A. baumannii ATCC 17978 strain and the clinical isolate NCCP 16007 are available in the NCBI database (accession numbers CP000521 and CP091465, respectively). 35 clinical isolates of A. baumannii, including the NCCP 16007 strain, were collected from the National Culture Collection for Pathogens, South Korea. All of the A. baumannii strains included in the study were cultured at 23°C or 37°C in Luria-Bertani (LB) broth with constant shaking at 220 rpm and aeration. All of the strains were cultured overnight (O/N) for 16 h. Following this, each O/N cell culture was diluted 1/100, and additional incubation was performed to ensure that all of the experiments were conducted in the mid-exponential phase (OD_600_ of approximately 0.5). Blue light treatments were performed using a custom LED lamp that emitted light at an intensity of 0 to 80 W/m^2^, with the emission peaks ranging from 400 to 470 nm and centered at 462 nm (14, 51, 52). The wavelength was determined using a BLACK-Comet-SR Spectrometer (StellarNet, USA) with SpectraWiz Spectroscopy software (StellarNet) and SpectraWiz LabVIEW Software (StellarNet). Blue light susceptibility tests were performed on LB agar plates inoculated with 10^7^ CFU/mL of each strain, with the light intensity ranging from 0 to 16 W/m^2^. The cell survival rate was calculated as the relative percentage in comparison with the number of colonies counted at 0 W/m^2^ (control). Statistical significance was determined using GraphPad Prism 9 (Student’s *t* test) for the comparison of the blue light susceptibilities between ATCC 17978 and NCCP 16007. Three biological replicates were used to validate our data.

10.1128/msystems.00897-22.7 TABLE S5 Bacterial strains used in the present study. ATCC, American Type Culture Collection; NCCP, National Culture Collection for Pathogens (Korea Disease Control and Prevention Agency [KDCA], Republic of Korea). Download Table S5, DOCX file, 0.02 MB. Copyright © 2023 Yang et al. 2023 Yang et al. https://creativecommons.org/licenses/by/4.0/ This content is distributed under the terms of the Creative Commons Attribution 4.0 International license.

### Assessment of *in vivo* protein-protein interactions using the BACTH system.

The BACTH system was used to assess the interactions between BlsA and BipA (53). As BlsA interacts with Fur in A. baumannii, Fur was used as a positive control for the BACTH system. Moreover, *fur* (473 bp) and *bipA* (363 bp) were cloned into pKNT25 and pKT25 vectors to fuse each protein into the adenylate cyclase subunit T25 of Bordetella pertussis. The plasmids and primers used in the BACTH system are listed in Tables S6 and S7, respectively. Each target gene (*fur* and *bipA*) was individually subcloned into the vector pKNT25 using the enzymes BamHI and HindIII to create *fur-T25* and *bipA-T25* fusion constructs, respectively, which had the adenylate cyclase subunit T25 at the C-terminal regions of the fused proteins. To create the *T25*-*bipA* fusion construct, *bipA* was subcloned into the vector pKT25, using the enzymes BamHI and EcoRI. The other subunit of adenylate cyclase, T18, was fused to BlsA in the pUT18C vector using the enzymes EcoRI and BamHI to produce the *T18-blsA* fusion protein. The *cyaA*-deleted strain E. coli BTH101 was cotransformed with the constructed plasmids pUT18C::*blsA* and pKNT25::*bipA*/*fur* as well as pUT18C::*blsA* and pKT25::*bipA*. Successful insertions were confirmed via the polymerase chain reaction (PCR) amplification and sequencing of the corresponding parts of the clones. The transformants were selected on LB agar plates supplemented with 5-bromo-4-chloro-3-indolyl-β-d-galactopyranoside (IPTG, 40 μg/mL), isopropyl-β-d-thiogalactopyranoside (X-Gal, 0.5 mM), ampicillin (100 μg/mL), and kanamycin (50 μg/mL). Colonies that were blue in color after being grown on the agar plate for 24 h were considered to have positive interactions. The β-galactosidase activity in LB broth was then assessed to quantify the interactions. Fivefold higher galactosidase activity (expressed in Miller units) than the negative control was considered to be a positive interaction (34, 35, 54). Statistical significance was determined using GraphPad Prism 9 (Student’s *t* test). Three biological replicates were used to validate the data. The cotransformation of pKNT25::*fur* and pUT18C::*blsA* into E. coli BTH101 was performed in the positive control. All of the tests were performed at least thrice at 23°C under blue light (4 W/m^2^) or dark conditions for 24 h.

10.1128/msystems.00897-22.8 TABLE S6 Plasmids used in this study. (The reference used: 53, 62, 64). Download Table S6, DOCX file, 0.02 MB. Copyright © 2023 Yang et al. 2023 Yang et al. https://creativecommons.org/licenses/by/4.0/ This content is distributed under the terms of the Creative Commons Attribution 4.0 International license.

10.1128/msystems.00897-22.9 TABLE S7 Primers used in this study. Download Table S7, DOCX file, 0.02 MB. Copyright © 2023 Yang et al. 2023 Yang et al. https://creativecommons.org/licenses/by/4.0/ This content is distributed under the terms of the Creative Commons Attribution 4.0 International license.

### Synteny analysis of *blsA* and homology modeling of BlsA.

Genomic assessments of *blsA* and its neighboring genes in Acinetobacter species were performed using the MaGe software package (55). Coding sequences (CDSs) were aligned with the corresponding CDSs in the Acinetobacter baumannii ATCC 17978 strain. The GC ratio was measured using the GC Content Calculator web tool (https://www.vectorbuilder.kr/tool/gc-content-calculator.html). Local sequence complexity and hydrophobicity analyses (transmembrane region of >1.8) of BlsA and BipA were performed using CLC Genomics Workbench v.10.0.1 (Qiagen, Germany). Homology modeling of BlsA (PDB entry 6W6Z, 100% sequence identity) and BipA (PDB entry 1NNX, 42% sequence identity) was initially performed using Phyre^2^ and was refined without ligands using GalaxyWEB software (56, 57). Each ligand was fitted in the ordered electron density using 3DLigandSite (58). All of the final products were visualized using the CLC Genomics Workbench v.10.0.1 (Qiagen). The amino acid sequences of OmpA in 360 genomes of Acinetobacter species were directly aligned over equalized lengths using ClustalW and were visualized using CLC Genomics Workbench v.10.0.1 (Qiagen). The DNA sequences of BlsA, 16S rDNA, and OmpA were aligned using ClustalW and were then displayed using the MEGA-X software package (59). Phylogenetic trees were constructed based on the distance matrices using the maximum-likelihood method and the Tamura-Nei model. Tree distances were calculated on the basis of the branch score distance with TreeDist in PHYLIP v3.695, according to a previous study (60, 61).

### RNA-seq-based transcriptome analysis and quantitative reverse transcription-PCR.

Total RNA was extracted from mid-exponentially grown ATCC 17978 cells (OD_600_ of approximately 0.5) using an RNeasy Mini Kit (Qiagen), according to the manufacturer’s instructions. The cells were incubated for 12 h under blue light (4 W/m^2^) or dark conditions in Luria-Bertani (LB) media (10 mL) at 23°C. All procedures for RNA sequencing were performed by DNA Link, Inc. (Republic of Korea). The relative transcript abundances are presented as fragments per kilobase of exon per million mapped sequence reads (FPKM). Genes exhibiting fold changes (FPKM values of blue light-treated cells/FPKM values of control cells) of greater than 1.5 and less than 0.5 were regarded as upregulated and downregulated genes, respectively. Genes with FPKM values of >50 in all transcriptome data were used in our gene expression analysis. All relevant RNA-seq data were deposited in the NCBI database under the Gene Expression Omnibus (GEO) accession numbers GSM5842295 (dark) and GSM5842296 (blue light). The expression levels of the target genes were normalized to the expression level of 16S rDNA, as described previously (62). Total RNAs from different A. baumannii strains were isolated in the mid-exponential phase (OD_600_ of approximately 0.5) under blue light (4 W/m^2^) or dark conditions in LB media (10 mL) using an RNeasy Mini Kit (Qiagen). For the qRT-PCR, cDNA was synthesized from the total extracted RNA (3 μg each) and amplified using the primers (qRT-PCR primers) listed in Table S7. Statistical significance was determined using GraphPad Prism 9 (Student’s *t* test). Three biological replicates were used to validate the data.

### Genetic manipulation of the A. baumannii strains.

*blsA-* and *bipA*-knockout mutants in the ATCC 17978 strain were constructed via single-crossover homologous recombination using pVIK112, as described previously (62). The primers used to amplify *blsA* and *bipA* are listed in Table S7. To construct the *blsA-* and *bipA*-knockout mutants, the broad-host-range suicide vector pVIK112 was digested with the restriction enzymes EcoRI and KpnI. The fragments of *blsA* and *bipA* were obtained via PCR amplification (*blsA*, 352 bp; *bipA*, 334 bp) and gel extraction. Then, the fragments were inserted into the pVIK112 vector via ligation. The fragment target gene-ligated pVIK112 vectors were then used to transform E. coli S17-1λ-*pir*. The extracted plasmids from the E. coli S17-1λ-*pir* cells were used to transform the ATCC 17978 strain via electroporation. The Campbell-type integration of our constructed suicide plasmids created gene crossover-knockout strains (63). The pEAb plasmid was constructed using the pSGAb-km (cat. no 121999, Addgene, USA) and pCasAb-apr (cat. no 121998, Addgene) plasmids. The kanamycin resistance gene (*aph(3′)-Ia*, aminoglycoside O-phosphotransferase) from the pSGAb-km plasmid was replaced with the apramycin resistance gene (*aac(3)-IV*, aminoglycoside 3-N-acetyltransferase type IV) from the pCasAb-apr plasmid for the successful selection of the MDR strain NCCP 16007. The *blsA-* and *bipA*-introduced pEAb plasmids were transformed into the NCCP 16007 strain to create the NCCP 16007/pEAb::*blsA* and NCCP 16007/pEAb::*bipA* strains, respectively. The corresponding genes, including their cognate promoter sites, were amplified from the chromosome of the ATCC 17978 strain. The transformation of both genes (*blsA* and *bipA*) into the NCCP 16007 strain (NCCP 16007/pEAb::*bipA*::*blsA*) was performed by inserting the whole genes from the chromosome of the ATCC 17978 strain individually into the pEAb vector, using the *bipA*_KO_F (EcoRI)/*blsA*_KO_R (KpnI) primer pair for gene amplification.

### CRISPR/Cas9-based knockout of *ompA* in A. baumannii.

The *ompA*-knockout mutant of the ATCC 17978 strain was constructed using the CRISPR-Cas9 system (64). A pair of a 20 bp spacer and an 80 nt single-stranded DNA (ssDNA) was designed to target the genomic locus and donor repair template for recombination, using the RGEN tool (http://www.rgenome.net/) (65). The 20 bp spacer oligonucleotides were phosphorylated and annealed to the pSGAb-km plasmid via a Golden Gate assembly reaction. Then, the plasmids were transformed into E. coli DH5α-electrocompetent cells, and this was followed by plating onto an LB agar plate with kanamycin (Kan, 50 μg/mL) and incubation at 37°C for 16 h. Successful cloning of the spacer was verified by PCR with the primer *ompA*_spacer_F and the universal primer M13R (5′-CAGGAAACAGCTATGACC-3′) as well as by sequencing the target construct with the primer M13R. To induce the expression of the RecAb recombination system and Cas9 nuclease, isopropyl β-d-1-thiogalactopyranoside (IPTG, 1 M) was added to the ATCC 17978 cells harboring the pCasAb-apr plasmid. After incubating at 37°C for 2 h, the cells were briefly washed with distilled water and were used to prepare electrocompetent cells, as described previously (64). Then, the spacer-cloned pSGAb-km plasmid (200 ng) and the 80 nt ssDNA (300 μM) were used to cotransform the IPTG-induced A. baumannii ATCC 17978 cells harboring the pCasAb-apr plasmid via electroporation. Recombinant cells were plated onto an LB agar plate containing apramycin (Apr, 100 μg/mL) and Kan (50 μg/mL), and they were then incubated at 37°C overnight (O/N). Successful gene editing was verified by PCR amplification and by the sequencing of the target regions. For plasmid curing, the freshly transferred cells were plated onto an LB agar plate containing sucrose (5% [wt/vol]) at 37°C O/N, and they were then streaked onto LB agar plates without the antibiotics to confirm the SacB counterselection. The growth of the colonies only on the antibiotic-free LB agar plate indicated that both the pCasAb-apr plasmid and the pSGAb-km plasmid were successfully cured in the colonies. The schematic overview of the genome editing is provided in Fig. 4A.

### Western blot analysis of the OmpA protein.

To verify our *ompA* deletion mutant, the ATCC 17978 and the *ompA* mutant were grown O/N at 37°C, and a further 1/100 dilution of O/N cultured cells in LB media with additional incubation was performed to ensure that all of our experiments were conducted in the mid-exponential phase (OD_600_ of approximately 0.5) with shaking at 23°C under blue light (4 W/m^2^) or dark conditions. Each cell culture was centrifuged at 8,000 rpm for 5 min and was washed twice with phosphate-buffered saline (PBS). Cell lysates were extracted using radio-immunoprecipitation assay (RIPA) buffer (25 mM Tris-HCl [pH 7.6], 150 mM NaCl, 1% [vol/vol] Nonidet P-40, 0.1% [wt/vol] sodium deoxycholate, 0.1% [wt/vol] SDS) and loaded via SDS-PAGE with 12% separating gel. Proteins were transferred to a polyvinylidene difluoride (PVDF) transfer membrane (Thermo Fisher Scientific, USA), and this was followed by blocking with skimmed milk (5% [wt/vol]) for 1 h. The proteins-transferred membranes were incubated with rabbit anti-OmpA antibody (CUSABIO, USA) O/N at 4°C. Subsequently, the secondary antibody was incubated at room temperature for 1 h, using the goat anti-rabbit antibody (Bio-Rad, USA). The signal was generated by horseradish peroxidase substrate (1 mL, Abfrontier, Republic of Korea) added on the membrane. The membrane was incubated at RT for 1 min, and immediate signal detection was conducted by using an iBright FL1500 imaging system (Thermo Fisher Scientific, USA). The broad range anti-GAPDH (ABCAM, Germany) was used as the loading control, according to a previous study (66). A densitometric analysis was performed using ImageJ software. Statistical significance was determined using GraphPad Prism 9 (Student’s *t* test). Three biological replicates were used to validate the data.

### Determination of ethidium bromide (EtBr) and meropenem influx.

The uptake of EtBr and meropenem was assessed in the ATCC 17978 and NCCP 16007 strains (36). Each strain was incubated in LB media at 37°C O/N and was diluted 100-fold with shaking at 23°C under blue light (4 W/m^2^) or dark conditions. Cells were harvested by centrifugation at room temperature and were washed twice with K-phosphate buffer (50 mM, pH 7) via centrifugation at 7,800 rpm for 5 min at room temperature. The final amount of cells (OD_600_ of approximately 0.5) was added to the same K-phosphate buffer (5 mL) that contained the proton conductor carbonyl cyanide 3-chlorophenylhydrazone (CCCP, 100 μM), which acts as an energy uncoupler and collapses the membrane energy involved in the efflux process (65). After the addition of EtBr (6 μM), the fluorescence of the EtBr-nucleic acid complex generated by the influx of EtBr into the cells was determined at room temperature using a Spark microplate reader (TECAN, Switzerland) at excitation and emission wavelengths of 515 and 590 nm, respectively. The uptake rates of meropenem through the outer membrane were calculated by measuring the concentration of meropenem in the supernatant. Bacteria were grown in LB media (5 mL) O/N at 37°C, and the culture (1.5 mL) was diluted in LB media (50 mL) that contained MgCl_2_ (5 mM). The cells were grown at 23°C for 12 h with aeration by shaking under blue light or dark conditions and were washed once with potassium phosphate buffer (pH 7.0, 50 mM) containing MgCl_2_ (5 mM). The washed cells were resuspended in the same buffer and were adjusted to the final amount (OD_600_ of approximately 0.5). Then, 3 mL of the sample was taken for the meropenem uptake assay. The sample (3 mL) was sonicated at a 5× duty cycle with a 15 s/burst, an interval of 30 s between bursts, and an output of 50%. The sonicated sample was used as a negative control for assessing the β-lactamase-mediated degradation and cellular binding of meropenem (10 μM). The lysates were centrifuged at 15,000 rpm in a microcentrifuge for 1 min, and the remaining meropenem concentration was measured. The reduction of the meropenem concentration in the sample was measured (297 nm) using a Spark microplate reader (TECAN) (67). The permeability coefficient [*P*, cm/s per 10^6^ cells] was calculated using the following equation:

$$P=C_{\mathrm{cells}}/(C_{\mathrm{lysate}}\;-\;C_{\mathrm{cells}}),$$

where *C_cells_* is the reduction rate of meropenem in the sample and *C_lysate_* is the reduction rate of meropenem in the negative control. Statistical significance was determined using GraphPad Prism 9 (Student’s *t* test). Three biological replicates were used to validate the data.

### Antibiotic susceptibility tests and the determination of the fractional inhibitory concentration index (FICI) under blue light.

The MICs of meropenem were determined with the broth-dilution method, using 96-well microtiter plates (SPL Life Science, Republic of Korea) (68). The FICI was determined via checkerboard analyses (69). In brief, each well of a 96-well plate was filled with LB broth (200 μL), and meropenem was 2-fold diluted along the abscissa from 0 to 128 μg/mL. Moreover, the intensity of blue light was adjusted to increase along the ordinate from 0 to 80 W/m^2^. The ATCC 17978 and NCCP 16007 strains were grown at 37°C in LB broth, diluted 100-fold, and incubated under blue light or dark conditions at 23°C or 37°C. Cells were harvested until each culture reached the early exponential phase (OD_600_ of approximately 0.5), and each well was inoculated with 10^6^ CFU of cells. The absorbance of each well at 600 nm was assessed after incubation at 23°C or 37°C for 30 h. The FICI (*ΣFIC*) was calculated for each well using the following equation:

$$\Sigma \mathrm{FIC}\;=\;\mathrm{FI}C^{A}\;+\;\mathrm{FI}C^{B}=\;\left(\mathrm{BL}\;\mathrm{intensit}y^{a}/\mathrm{BL}\;\mathrm{intensit}y^{b}\right)\;+\;\left(\mathrm{MP}N^{a}/\mathrm{MP}N^{b}\right),$$

where *BL intensity^a^* is the MIC of blue light intensity (W/m^2^) in the presence of meropenem, *BL intensity^b^* is the MIC of blue light intensity (W/m^2^) in the absence of meropenem, *MPN^a^* is the MIC of meropenem (μg/mL) when treated under blue light, and *MPN^b^* is the MIC of meropenem (μg/mL) when treated under dark conditions. Synergy is defined as a FICI of ≤0.5 (70). Statistical significance was determined using GraphPad Prism 9 (Student’s *t* test) for the comparison of the *BL intensity^a^* versus the *BL intensity^b^* as well as the comparison of *MPN^a^* versus *MPN^b^*. Three biological replicates were used to validate the data.

### Production of H_2_O_2_ and phenotypic analyses.

All of the tested samples were incubated at 23°C under blue light (400 to 470 nm, 4 W/m^2^) or dark conditions. The production of H_2_O_2_ was measured during the cultivation of the ATCC 17978 and NCCP 16007 strains in PhotoBiobox at light intensities of 0 (dark) and 4 W/m^2^ (71). All of the cells were incubated in 96-well plates (200 μL). After incubation for 30 h, the amount of H_2_O_2_ in the filtered samples was measured using the Amplex Red Hydrogen Peroxide/Peroxidase Assay Kit (Thermo Fisher Scientific, USA). To quantify biofilm production, mid-exponentially grown cells (OD_600_ of approximately 0.5, 10^6^ CFU) were inoculated into LB broth (200 μL/well) in a 96-well plate. LB broth with no cells was used as a negative control. The microtiter plates were then incubated for 30 h. After removing planktonic cells, the biofilm biomass was stained with crystal violet and was solubilized with 95% ethanol (vol/vol). Following this, the absorbance was measured at 595 nm. For the scanning electron microscopic (SEM) observations, O/N incubated cells in LB broth were diluted 1/100 in fresh LB broth, and additional incubation was performed in a 15 mL glass test tube with/without blue light irradiation (4 W/m^2^) at 23°C for 12 h, until each strain reached its mid-exponential phase (OD_600_ of approximately 0.5). Cells (l mL) were harvested to prepare for analysis and were first fixed with low-strength Karnovsky’s solution (paraformaldehyde [2% vol/vol], glutaraldehyde [2.5% vol/vol], and phosphate buffer [0.1 M; final pH, 7.2]) for 4 h. Secondary fixation was done using an osmium tetroxide solution (2% [vol/vol]) at 4°C for 2 h. The fixed samples were gradually dehydrated with ethanol (30%, 50%, and 70%) for 10 min each, and they were placed on an aluminum stub O/N to dry at room temperature. These samples were then coated with platinum and were assessed via a FE-SEM analysis (Quanta 250 FEG; FEI, USA). Statistical significance was determined using GraphPad Prism 9 (Student’s *t* test). Three biological replicates were used to validate the data.

### Data availability.

The authors confirm that the data supporting the findings of the present study are available within the article and/or its supplemental materials. The data sets generated during the study are available in the NCBI database under the Gene Expression Omnibus (GEO) accession numbers GSM5842295 and GSM5842296.
