# Supplementary figures and images for: Blue Light Sensing BlsA-Mediated Modulation of Meropenem Resistance and Biofilm Formation in Acinetobacter baumannii
Source: mSystems. 2023 Jan 9;8(1):e00897-22. doi: 10.1128/msystems.00897-22 (PMC9948694; doi:10.1128/msystems.00897-22)

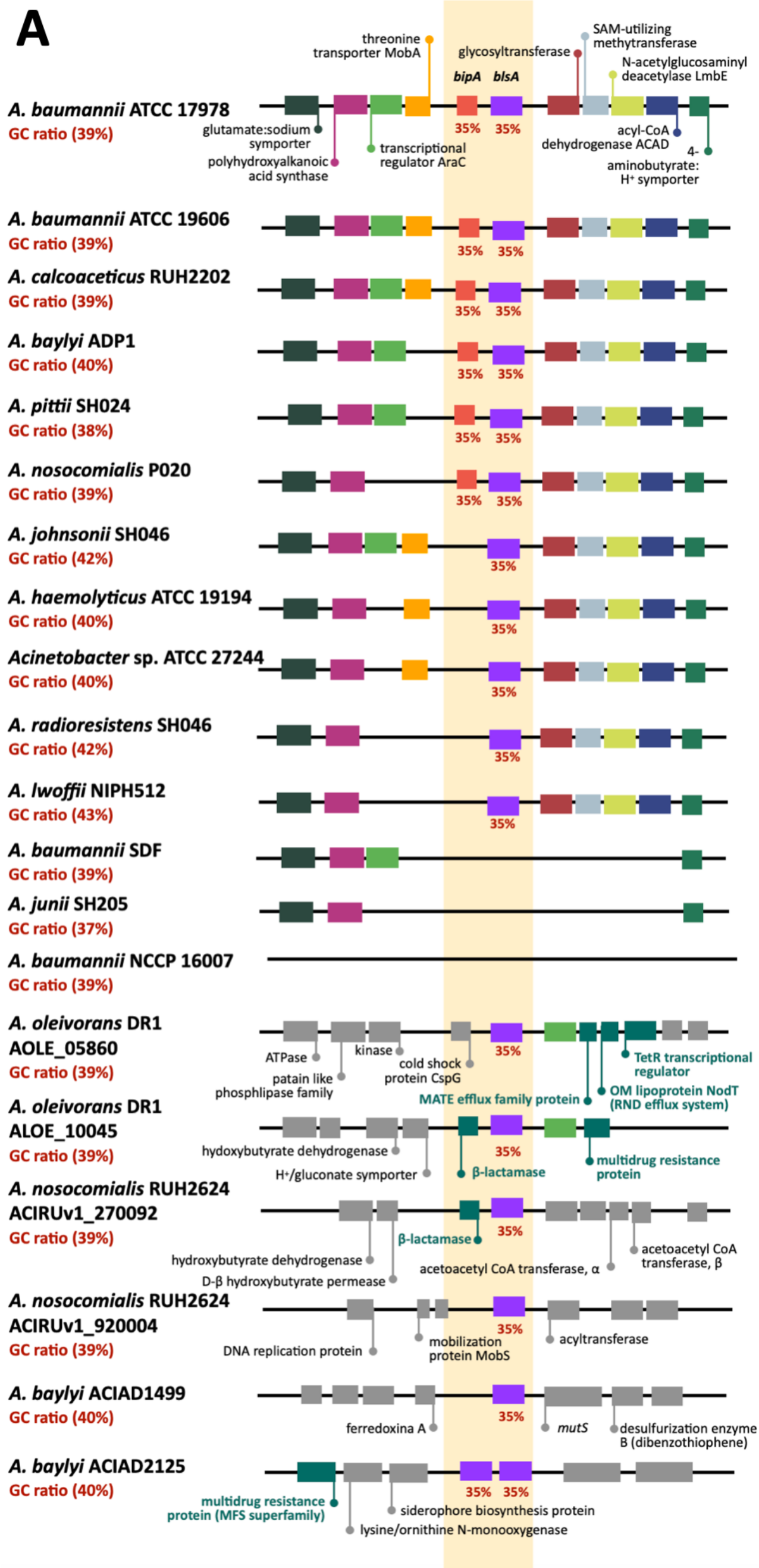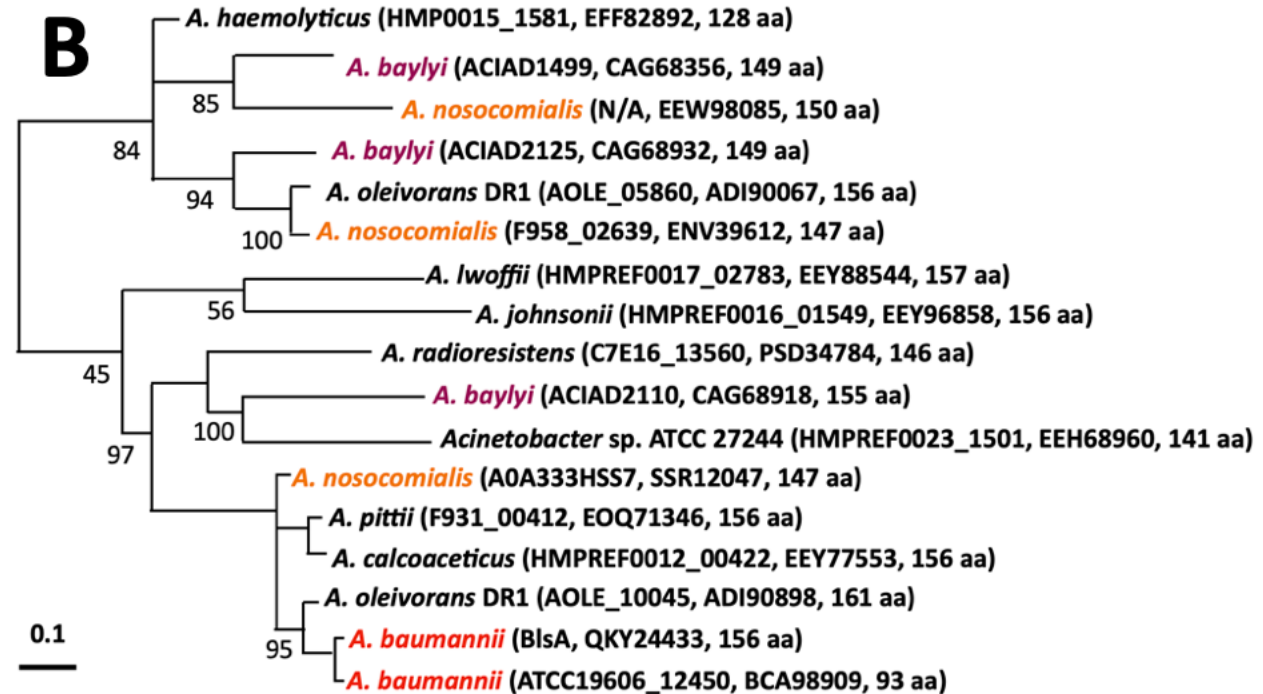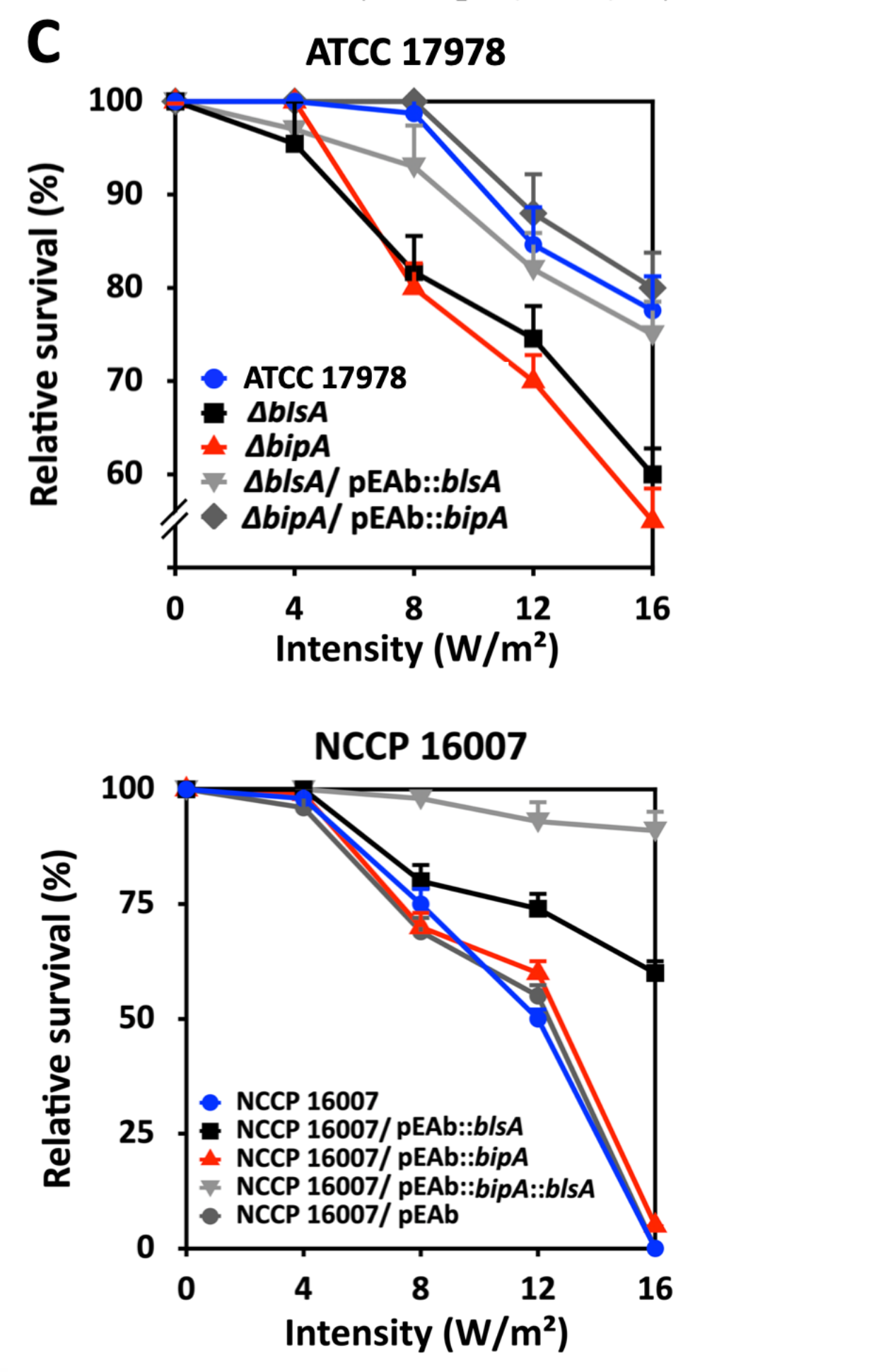

Supplement: FIG S1 [file msystems.00897-22-s0001.pdf]

**A**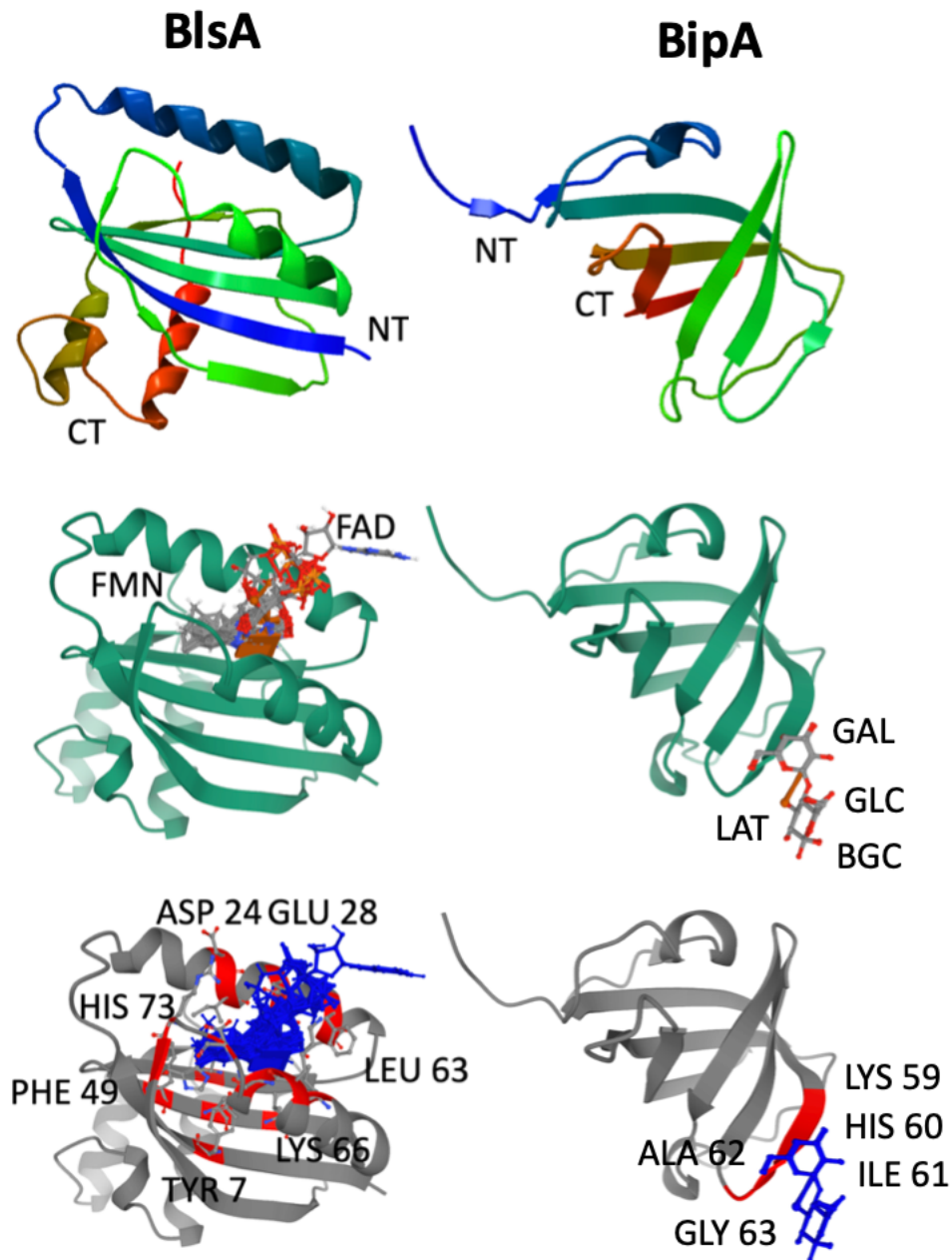**B**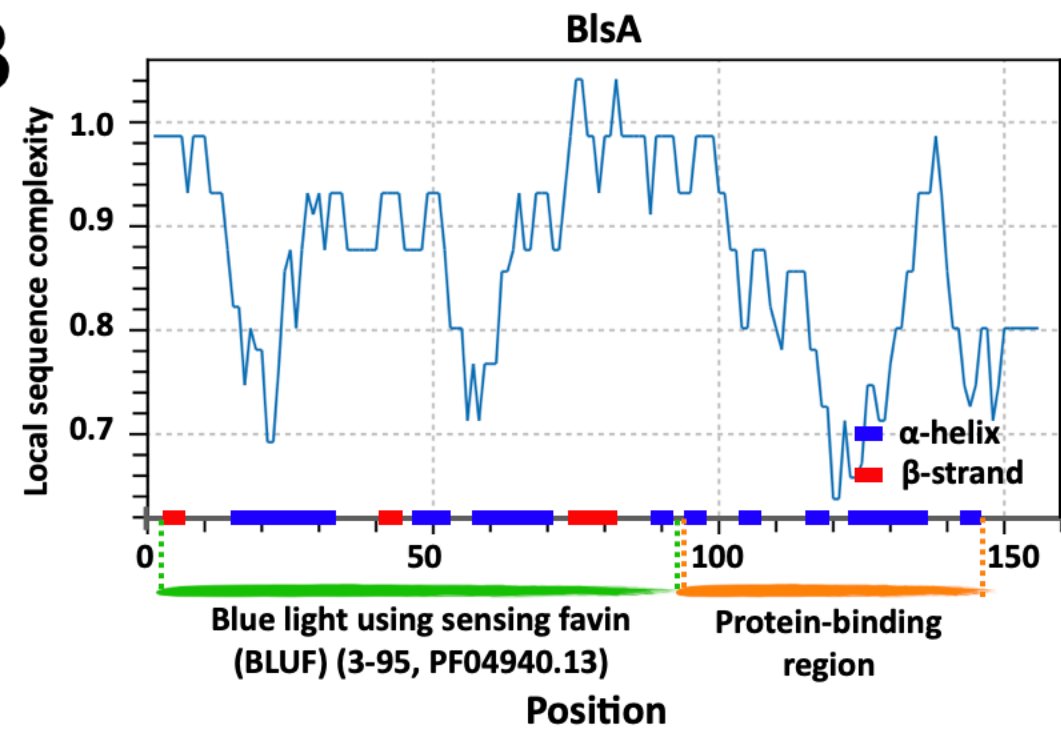**C**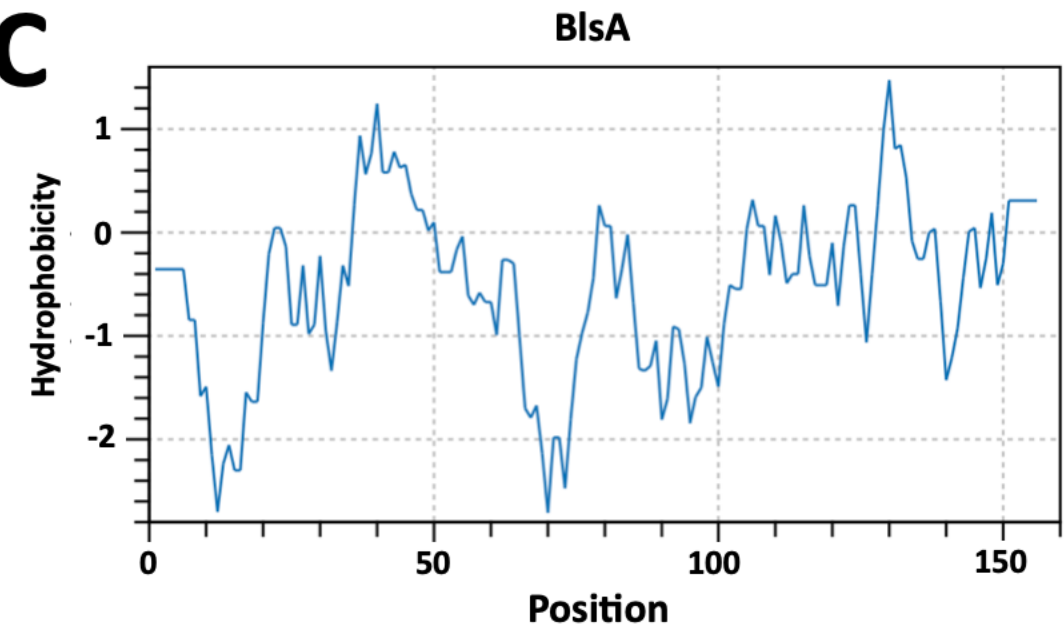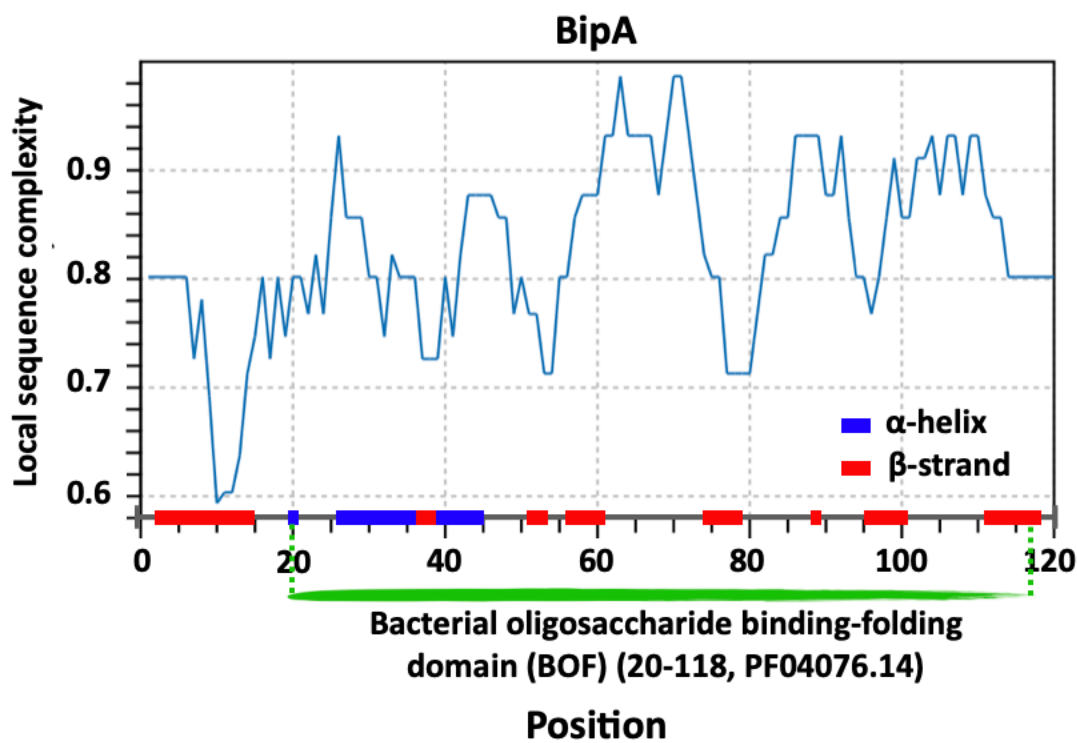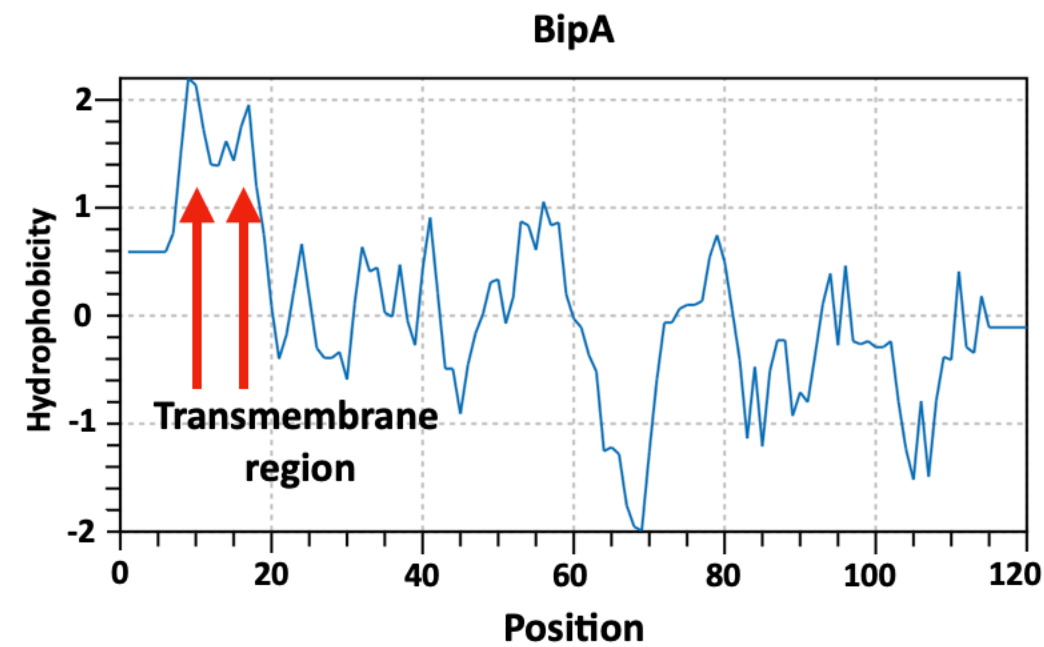

Supplement: FIG S2 [file msystems.00897-22-s0002.pdf]
