# Supplementary material for: Blue Light Sensing BlsA-Mediated Modulation of Meropenem Resistance and Biofilm Formation in Acinetobacter baumannii
Source: mSystems. 2023 Jan 9;8(1):e00897-22. doi: 10.1128/msystems.00897-22 (PMC9948694; doi:10.1128/msystems.00897-22)
Supplement: TABLE S1 [file msystems.00897-22-s0003.docx]

**Table S1.** Light susceptibilities of clinical *A. baumannii* isolates.

| **Isolate** | ***blsA**** | ***bipA**** | **BL25 (**W/ m^2^) | **BL50 (**W/ m^2^) | **Origin** | **Region**** | |
| --- | --- | --- | --- | --- | --- | --- | --- |
| NCCP 15995 | A | A | 4 | 8 | N/A | SU |  |
| NCCP 16009 | A | A | 4 | 8 | N/A | SU |  |
| NCCP 16006 | A | A | 4 | 8 | N/A | SU |  |
| NCCP 16007 | A | A | 8 | 12 | urine | SU |  |
| NCCP 16004 | A | A | 8 | 12 | urine | SU |  |
| NCCP 16008 | A | A | 8 | 12 | N/A | SU |  |
| NCCP 16011 | A | A | 8 | 12 | N/A | SU |  |
| ATCC 17978 | P | P | ≥16 | ≥16 | Laboratory |  |  |
| NCCP 16002 | P | P | ≥16 | ≥16 | sputum | GW |  |
| NCCP 12276 | P | P | ≥16 | ≥16 | other | BS |  |
| NCCP 12277 | P | P | ≥16 | ≥16 | Pus | GJ |  |
| NCCP 12278 | P | P | ≥16 | ≥16 | sputum | GN |  |
| NCCP 16001 | P | P | ≥16 | ≥16 | other | CB |  |
| NCCP 15988 | P | P | ≥16 | ≥16 | sputum | JN |  |
| NCCP 15999 | P | P | ≥16 | ≥16 | sputum | GN |  |
| NCCP 14608 | P | P | ≥16 | ≥16 | pus | DG |  |
| NCCP 16000 | P | P | 16 | ≥16 | prostate | GG |  |
| NCCP 15996 | P | P | 16 | ≥16 | urine | GG |  |
| NCCP 15987 | P | P | 16 | ≥16 | Pus | IC |  |
| NCCP 14654 | P | P | 16 | ≥16 | other | GN |  |
| NCCP 14782 | P | P | 16 | ≥16 | pus | GN |  |
| NCCP 15989 | P | P | 16 | ≥16 | sputum | JB |  |
| NCCP 14607 | P | P | 16 | ≥16 | sputum | DG |  |
| NCCP 15990 | P | P | 12 | 16 | sputum | GB |  |
| NCCP 15998 | P | P | 12 | 16 | sputum | GB |  |
| NCCP 15994 | P | P | 12 | 16 | pus | GB |  |
| NCCP 14609 | P | P | 12 | 16 | pleural fluid | DG |  |
| NCCP 15997 | P | P | 12 | 16 | urine | JN |  |
| NCCP 15991 | P | P | 12 | 16 | urine | GB |  |
| NCCP 16005 | P | P | 12 | 16 | sputum | GB |  |
| NCCP 16003 | P | P | 12 | 16 | sputum | GN |  |
| NCCP 15992 | P | P | 12 | 16 | N/A | SU |  |
| NCCP 16010 | P | P | 12 | 16 | N/A | SU |  |
| NCCP 14655 | P | P | 12 | 16 | pus | GN |  |
| NCCP 14606 | P | P | 12 | 16 | cerebrospinal fluid | DG |  |
| NCCP 15993 | P | P | 8 | 12 | pus | SU |  |
