# Supplementary material for: Blue Light Sensing BlsA-Mediated Modulation of Meropenem Resistance and Biofilm Formation in Acinetobacter baumannii
Source: mSystems. 2023 Jan 9;8(1):e00897-22. doi: 10.1128/msystems.00897-22 (PMC9948694; doi:10.1128/msystems.00897-22)
Supplement: TABLE S2 [file msystems.00897-22-s0004.docx]

**Table S2.** List of genes that were upregulated or downregulated in the *A. baumannii* ATCC 17978 cells under blue light irradiation.

| **Gene symbol** | **Product** | **GC content (%)** | **TM*** | **Subcellular location** | **FAD/ NAD(P)-binding site*** | **FPKM** | | **Fold change** |
| --- | --- | --- | --- | --- | --- | --- | --- | --- |
|  |  |  |  |  |  | **Dark** | **Light** |  |
|  | **Up-regulated genes under blue-light** | | | | | | | |
|  | **Trehalose biosynthesis** | | | | | | | |
| *otsA* | Trehalose-6-phosphate synthase | 38 | - | Cytosol | - | 103.84 | 217.45 | 2.09 |
| *otsB* | Trehalose-phosphatase | 27 | - | Cytosol | - | 19.90 | 55.53 | 2.79 |
|  | **Catalytic activity** | | | | | | | |
| *SDR* | SDR family oxidoreductase | 38 | TM | Inner membrane | NAD | 24.56 | 78.03 | 3.18 |
| *gstA* | Glutathione S-transferase family protein | 40 | - | Cytosol | - | 117.24 | 321.87 | 2.75 |
| *hmp* | Flavohemoprotein | 38 | TM | Inner membrane | FAD | 54.97 | 140.16 | 2.55 |
| *phnB* | VOC family protein | 38 | TM | Inner membrane | - | 124.22 | 316.67 | 2.55 |
| *rnfC* | Electron transport complex protein RnfC | 35 | TM | Inner membrane | FAD | 459.94 | 1019.45 | 2.22 |
| *1-cys prx* | Peroxiredoxin | 38 | - | Cytosol | - | 115.03 | 241.63 | 2.10 |
| *ydjA* | Nitroreductase family protein | 38 | - | Cytosol | FMN | 59.14 | 104.14 | 1.76 |
| *katE* | Catalase HPII | 41 | TM | Cytosol | - | 798.48 | 1323.8 | 1.65 |
|  | **Stress response** | | | | | | | |
| *KGG* | Stress-induced bacterial acidophilic repeat motif | 46 | - | Cytosol | - | 32.85 | 287.30 | 8.75 |
| *bipA* | NirD/YgiW/Ydel family stress tolerance protein | 35 | TM | N/A | - | 59.04 | 169.29 | 2.87 |
| *blsA* | BLUF domain-containing protein | 35 | - | Cytosol | FAD | 55.10 | 83.28 | 1.512 |
|  | **Molecular chaperone** | | | | | | | |
| *papD* | Molecular chaperone | 32 | - | Periplasm | - | 19.29 | 52.67 | 2.73 |
|  | **Outermembrane porin** | | | | | | | |
| *ompA* | OmpA family protein | 40 | TM | Outer membrane | - | 139.23 | 327.76 | 2.35 |
| *carO* | Ornithine uptake porin CarO type IV | 39 | TM | Outer membrane | - | 1996.52 | 3444.60 | 1.73 |
| *omp33-36* | Porin Omp33-36 | 39 | TM | Outer membrane | - | 1419.24 | 2426.08 | 1.71 |
| *pxpA* | LamB/YcsF family protein | 44 | TM | Outer membrane | - | 48.50 | 79.15 | 1.63 |
| *aqpZ* | Aquaporin Z | 45 | TM | Outer membrane | - | 287.63 | 457.80 | 1.59 |
|  | **Metabolic process** | | | | | | | |
| *mmsA* | CoA-acylating methylmalonate-semialdehyde dehydrogenase | 43 | - | Cytosol | NAD | 13.09 | 70.32 | 5.37 |
| *atoD* | CoA transferase subunit A | 37 | - | Cytosol | - | 80.03 | 205.65 | 2.57 |
| *atoA* | CoA transferase subunit B | 40 | - | Cytosol | - | 47.42 | 116.78 | 2.46 |
| *ACAD* | Acyl-CoA dehydrogenase family protein | 44 | - | Cytosol | FAD | 36.01 | 79.53 | 2.21 |
| *IVD* | Isovaleryl-CoA dehydrogenase | 43 | - | Cytosol | - | 91.49 | 197.10 | 2.15 |
|  | **Translation, ribosomal structure and biogenesis** | | | | | | | |
| *mnmG* | tRNA U34 5-carboxymethylaminomethyl modifying enzyme MnmG/GidA | 35 | - | Cytosol | - | 179.35 | 423.46 | 2.36 |
|  | **Down-regulated genes under blue-light** | | | | | | | |
| *metE* | Methionine synthase | 43 | - | Cytosol | - | 531.46 | 158.34 | 0.30 |
| *snaC* | Flavin reductase | 43 | - | Cytosol | FAD | 176.51 | 82.17 | 0.47 |
| *cydA* | Cytochrome ubiquinol oxidase subunit I | 41 | TM | Inner membrane | - | 617.83 | 303.21 | 0.49 |
| *cydB* | Cytochrome d ubiquinol oxidase subunit II | 41 | TM | Inner membrane | - | 746.92 | 373.46 | 0.50 |
| *cydX* | Cytochrome bd-I oxidase subunit CydX | 39 | TM | Inner membrane | - | 26526.30 | 0 | - |
| *ybgE* | Cyd operon YbgE family protein | 44 | TM | Inner membrane | - | 248.60 | 93.57 | 0.38 |
