# Supplementary material for: Blue Light Sensing BlsA-Mediated Modulation of Meropenem Resistance and Biofilm Formation in Acinetobacter baumannii
Source: mSystems. 2023 Jan 9;8(1):e00897-22. doi: 10.1128/msystems.00897-22 (PMC9948694; doi:10.1128/msystems.00897-22)
Supplement: TABLE S3 [file msystems.00897-22-s0005.docx]

**Table S3.** Determination of meropenem uptake rates.

| Strain | Condition | Cells* | Lysate* | Permeability coefficient** |
| --- | --- | --- | --- | --- |
| ATCC 17978 | BL | 1.0 × 10^0^ | 6.1 × 10^-1^ | 1.1 × 10^0^ |
|  | DK | 2.3 × 10^-1^ | 2.7 × 10^-1^ | 2.6 × 10^-1^ |
| *∆ompA* | BL | 1.2 × 10^-1^ | 2.5 × 10^-1^ | 1.5 × 10^-1^ |
|  | DK | 1.3 × 10^-1^ | 2.1 × 10^-1^ | 1.3 × 10^-1^ |
| *∆blsA* | BL | 1.9 × 10^-1^ | 2.4 × 10^-1^ | 1.2 × 10^-1^ |
|  | DK | 1.7 × 10^-1^ | 2.4 × 10^-1^ | 2.0 × 10^-1^ |
| *∆bipA* | BL | 2.1 × 10^-1^ | 3.2 × 10^-1^ | 2.4 × 10^-1^ |
|  | DK | 1.7 × 10^-1^ | 2.3 × 10^-1^ | 0.2 × 10^-1^ |
| *∆blsA*/ pEAb::*blsA* | BL | 1.0 × 10^0^ | 1.3 × 10^-1^ | 1.1 × 10^0^ |
|  | DK | 2.0 × 10^-1^ | 2.2 × 10^-1^ | 1.7 × 10^-1^ |
| *∆bipA*/ pEAb::*bipA* | BL | 1.1 × 10^0^ | 5.3 × 10^-1^ | 1.2 × 10^-0^ |
|  | DK | 2.1 × 10^-1^ | 2.3 × 10^-1^ | 2.4 × 10^-1^ |
| NCCP 16007 | BL | 5.9 × 10^-1^ | 2.8 × 10^-1^ | 2.1 × 10^-1^ |
|  | DK | 7.0 × 10^-2^ | 2.7 × 10^-1^ | 1.0 × 10^-1^ |
| NCCP 16007/ pEAb::*blsA* | BL | 6.6 × 10^-1^ | 2.2 × 10^-1^ | 7.3 × 10^-1^ |
|  | DK | 7.8 × 10^-2^ | 3.0 × 10^-1^ | 1.2 × 10^-1^ |
| NCCP 16007/ pEAb::*bipA* | BL | 9.9 × 10^-2^ | 2.6 × 10^-1^ | 1.2 × 10^-1^ |
|  | DK | 8.9 × 10^-2^ | 2.7 × 10^-1^ | 1.1 × 10^-1^ |
| NCCP 16007/ pEAb::*bipA*::*blsA* | BL | 1.5 × 10^0^ | 2.2 × 10^-1^ | 1.7 × 10^0^ |
|  | DK | 6.7 × 10^-2^ | 2.4 × 10^-1^ | 1.0 × 10^-1^ |
| NCCP 16007/ pEAb | BL | 2.3 × 10^-1^ | 2.8 × 10^-1^ | 2.6 × 10^-1^ |
|  | DK | 9.0 × 10^-2^ | 2.8 × 10^-1^ | 1.2 × 10^-1^ |
