# Supplementary material for: Blue Light Sensing BlsA-Mediated Modulation of Meropenem Resistance and Biofilm Formation in Acinetobacter baumannii
Source: mSystems. 2023 Jan 9;8(1):e00897-22. doi: 10.1128/msystems.00897-22 (PMC9948694; doi:10.1128/msystems.00897-22)
Supplement: TABLE S4 [file msystems.00897-22-s0006.docx]

**Table S4.** Synergistic activity of meropenem with blue light against *A. baumannii* strains.

| Strain (condition) | BL intensity^a^ | BL intensity^b^ | MPN^a^ | MPN^b^ | FICI |
| --- | --- | --- | --- | --- | --- |
| ATCC 17978 (23°C) | 20 | 80* | 0.25 | 1^#^ | 0.50 |
| ATCC 17978 (37°C) | 60 | 80 | 0.5 | 1 | 1.25 |
| *∆ompA* (23°C) | 80 | 80 | 2 | 2 | 2.00 |
| *∆ompA* (37°C) | 80 | 80 | 2 | 2 | 2.00 |
| *∆blsA* (23°C) | 12 | 16 | 0.5 | 1 | 1.25 |
| *∆blsA* (37°C) | 12 | 16 | 0.5 | 1 | 1.25 |
| *∆bipA* (23°C) | 12 | 16 | 0.5 | 1 | 1.25 |
| *∆bipA* (37°C) | 12 | 16 | 0.5 | 1 | 1.25 |
| *∆blsA*/ pEAb::*blsA* (23°C) | 20 | 80* | 0.25 | 1^#^ | 0.50 |
| *∆blsA*/ pEAb::*blsA* (37°C) | 60 | 80 | 0.5 | 1 | 1.25 |
| *∆bipA*/ pEAb::*bipA* (23°C) | 20 | 80* | 0.25 | 1^#^ | 0.50 |
| *∆bipA*/ pEAb::*bipA* (37°C) | 60 | 80 | 0.5 | 1 | 1.25 |
| NCCP 16007 (23°C) | 12 | 16 | 8 | 16 | 1.25 |
| NCCP 16007 (37°C) | 12 | 16 | 8 | 16 | 1.25 |
| NCCP 16007/ pEAb::*blsA* (23°C) | 20 | 80* | 4 | 16^#^ | 0.50 |
| NCCP 16007/ pEAb::*blsA* (37°C) | 60 | 80 | 8 | 16 | 1.25 |
| NCCP 16007/ pEAb::*bipA* (23°C) | 12 | 16 | 8 | 16 | 1.25 |
| NCCP 16007/ pEAb::*bipA* (37°C) | 12 | 16 | 8 | 16 | 1.25 |
| NCCP 16007/ pEAb::*bipA*::*blsA* (23°C) | 20 | 80* | 2 | 16^#^ | 0.38 |
| NCCP 16007/ pEAb::*bipA*::*blsA* (37°C) | 60 | 80 | 8 | 16 | 1.25 |
| NCCP 16007/ pEAb (23°C) | 12 | 16 | 8 | 16 | 1.25 |
| NCCP 16007/ pEAb (37°C) | 12 | 16 | 8 | 16 | 1.25 |
