# Supplementary material for: Blue Light Sensing BlsA-Mediated Modulation of Meropenem Resistance and Biofilm Formation in Acinetobacter baumannii
Source: mSystems. 2023 Jan 9;8(1):e00897-22. doi: 10.1128/msystems.00897-22 (PMC9948694; doi:10.1128/msystems.00897-22)
Supplement: TABLE S5 [file msystems.00897-22-s0007.docx]

**Table S5.** Bacterial strains used in the present study.

| **Strain** | **Description** | **Reference** |
| --- | --- | --- |
| ATCC 17978 | Wild-type, laboratory-cultured *A. baumannii* ATCC 17978 | Laboratory stock |
| Δ*blsA* | *blsA* (encoded by ATCC1_01506) mutant; insertion of pVIK112-*blsA* into ATCC 17978 | This study |
| Δ*bipA* | *bipA* (encoded by ATCC1_01505) mutant; insertion of pVIK112- *bipA* into ATCC 17978 | This study |
| Δ*blsA/* pEAb::*blsA* | Complementation strain; insertion of pEAb-*blsA,* into Δ*blsA* | This study |
| Δ*bipA/* pEAb::*bipA* | Complementation strain; insertion of pEAb-*bipA,* into Δ*bipA* | This study |
| NCCP 16007 | Clinical *A. baumannii* strain isolated from a patient with a urinary tract infection | (1) |
| NCCP 16007/ pEAb::*blsA* | *blsA-*transformed strain; insertion of pEAb-*blsA*, into NCCP 16007 | This study |
| NCCP 16007/ pEAb::*bipA* | *bipA-*transformed strain; insertion of pEAb-*bipA*, into NCCP 16007 | This study |
| NCCP 16007/ pEAb::*bipA*::*blsA* | *bipA*-*, blsA-*transformed strain; insertion of pEAb-*bipA*-*blsA*, into NCCP 16007 | This study |
| NCCP 16007/ pEAb | Vector-control strain; insertion of empty vector, pEAb, into NCCP 16007 | This study |

1. Kim M, Park J, Park W. 2021. Genomic and phenotypic analyses of multidrug-resistant *Acinetobacter baumannii* NCCP 16007 isolated from a patient with a urinary tract infection. *Virulence*. 12:150-164.
