# Supplementary material for: Blue Light Sensing BlsA-Mediated Modulation of Meropenem Resistance and Biofilm Formation in Acinetobacter baumannii
Source: mSystems. 2023 Jan 9;8(1):e00897-22. doi: 10.1128/msystems.00897-22 (PMC9948694; doi:10.1128/msystems.00897-22)
Supplement: TABLE S6 [file msystems.00897-22-s0008.docx]

**Table S6.** Plasmids used in this study.

| **Plasmids** | **Description** | **Reference** |
| --- | --- | --- |
| pUT18C | Amp^r^, internal T18 fragment (amino acids 225 to 399 of CyaA) in pUC19 | (1) |
| pUT18C::*blsA* | Amp^r^, internal *blsA* fragment (encoded by ATCC1_01506) in pUT18C | This study |
| pKT25 | Km^r^, internal T25 fragment (amino acids 1 to 224 of CyaA) in pSU40 | (1) |
| pKT25::*bipA* | Km^r^, internal *bipA* fragment (encoded by ATCC1_01505) in pKT25 | This study |
| pKNT25 | Km^r^, internal T25 fragment (amino acids 1 to 224 of CyaA) in pSU40 | (1) |
| pKNT25::*fur* | Km^r^, internal *fur* fragment (encoded by ATCC1_02963) in pKNT25 | This study |
| pKNT25::*bipA* | Km^r^, internal *bipA* fragment (encoded by ATCC1_01505) in pKNT25 | This study |
| pVIK112 | Km^r^, R6K, *oriV*, suicide vector, *lacZ* fusion | (2) |
| pCasAb-apr | Apr^r^, *oriV*, broad-host-range vector | (3) |
| pSGAb-km | Km^r^*, ColE1, WH1266* | (3) |
| pEAb | Apr^r^, internal *Apr* fragment (encoded by pCasAb-apr) in pSGAb-km | This study |
| pVIK112::*blsA* | Km^r^, internal *blsA* fragment (encoded by ATCC1_01506) in pVIK112 | This study |
| pVIK112::*bipA* | Km^r^, internal *bipA* fragment (encoded by ATCC1_01505) in pVIK112 | This study |
| pEAb::*blsA* | Apr^r^, internal *blsA* fragment (encoded by ATCC1_01506) in pEAb | This study |
| pEAb::*bipA* | Apr^r^, internal *bipA* fragment (encoded by ATCC1_01505) in pEAb | This study |
| pEAb::*bipA*::*blsA* | Apr^r^, internal *bipA* and *blsA* fragment (encoded by ATCC1_01505 and ATCC1_01506) in pEAb | This study |

1. Karimova G, Gauliard E, Davi M, Ouellette SP, Ladant D. 2017. Protein-protein interaction: bacterial two-hybrid. *Methods Mol Biol*. 1615:159–176.
2. Park J, Kim M, Shin B, Kang M, Yang J, Lee TK, Park W. 2021. A novel decoy strategy for polymyxin resistance in *Acinetobacter baumannii*. *eLife*. 10:e66988.
3. Wang Yu, Wang Z, ChenY, Hua X, Yu Y, Ji Q. 2019. A highly efficient CRISPR Cas9-based genome engineering platform in *Acinetobacter baumannii* to understand the H_2_O_2_-sensing mechanism of OxyR. *Cell Chem Biol.* 26:1732-1.
