# Supplementary material for: Blue Light Sensing BlsA-Mediated Modulation of Meropenem Resistance and Biofilm Formation in Acinetobacter baumannii
Source: mSystems. 2023 Jan 9;8(1):e00897-22. doi: 10.1128/msystems.00897-22 (PMC9948694; doi:10.1128/msystems.00897-22)
Supplement: TABLE S7 [file msystems.00897-22-s0009.docx]

**Table S7.** Primers used in this study.

| **BACTH primers** | **Sequence (5′-3′)** | **Reference** |
| --- | --- | --- |
| *blsA*_pUT18_F (*Hind*III) | AAGCTTGATGCCTATTTCCAATCAAGAT | This study |
| *blsA*_pUT18_R (*Bam*HI) | GGATCCTCTTTCTTGCGCAATGC | This study |
| *bipA*_pKT25_F (*Bam*HI) | GGATCCCATATTATTTACCACACTCAC | This study |
| *bipA*_pKT25_R (*Eco*RI) | GAATTCTTATTTAACACGTTCAATTTG | This study |
| *bipA*_pKNT25_F (*Hind*III) | CGCAAGCTTGATGAAAAAAATATTATTT | This study |
| *bipA*_pKNT25_R (*Bam*HI) | GGATCCTCTTGAGCTGCTGCAGGAG | This study |
| *fur* _pKNT25_F (*Hind*III) | AAGCTTGATGCCTATTTCCAATCAAGAT | This study |
| *fur* _pKNT25_R (*Bam*HI) | GGATCCTCTTTCTTGCGCAATGC | This study |
| **Knockout primers** | **Sequence (5′-3′)** | **Reference** |
| *blsA*_KO_F (*Eco*RI) | GAATTCCTGTGTTATGCCAGCCAACG | This study |
| *blsA*_KO_R (*Kpn*I) | GGTACCGGTTCAGCAAAAGCGGGTTA | This study |
| *bipA*_KO_F (*Eco*RI) | GAATTCGCAAGAACAGATCCCGCTTTAC | This study |
| *bipA*_KO_R (*Kpn*I) | GGTACCCTCCAACCTTTAACTGCCAACC | This study |
| **CRISPR/Cas9 primers** | **Sequence (5′-3′)** | **Reference** |
| *ompA_*spacer*_*F | TAGTACAGTTACTCCATTATTGCT | This study |
| *ompA_*spacer*_*R | AAACAGCAATAATGGAGTAACTGT | This study |
| *ompA_*ssDNA (80nt) | TGAGTCGTATTGCACTTGCTACTATGCTTGTTGCTGCTCCCGAAGCTGAATATAACCAAGTTAAAGGCGACGTAGACGGC | This study |
| *ompA_*sequencing_F | ATGAAATTGAGTCGTATTGCACT | This study |
| *ompA_*sequencing_R | TTGAGCTGCTGCAGGAG | This study |
| **Cloning primers** | **Sequence (5′-3′)** | **Reference** |
| *apar*_pEAb_F (*Eco*RI) | GAATTCTACCGTCGACCTCGAGGGG | This study |
| *apar*_pEAb_R (*Hind*III) | AAGCTTGGAATTGCCGGGCTATGTG | This study |
| *blsA*_pEAb_F (*Not*I) | GCGGCCGCATGAACGTTCGCCTGTGT | This study |
| *blsA*_pEAb_R (*Eco*RI) | GAATTCGAACGGGTTTACTCCCCG | This study |
| *bipA*_pEAb_F (*Not*I) | GCGGCCGCCGGGTAACTACCAGTAGTCG | This study |
| *bipA*_pEAb_R (*Eco*RI) | GAATTCTTATTTAACACGTTCAATTTG | This study |
| **qRT-PCR primers** | **Sequence (5′-3′)** | **Reference** |
| 16S_qRT PCR_F | TGGTGCCTTCGGGAATCTAG | This study |
| 16S_qRT PCR_R | TGCGGGACTTAACCCAACAT | This study |
| *blsA*_qRT PCR_F | TTATGCCAGCCAACGAAATG | This study |
| *blsA*_qRT PCR_R | ACAAATCCCGTTTAAATCGTTGA | This study |
| *ompA*_qRT PCR_F | ATTGCACTTGCTACTATGCTTGTTG | This study |
| *ompA*_qRT PCR_R | GGCTGTCTTGGAAAGTGTAACCA | This study |
| *bipA*_qRT PCR_F | AATCGTGATTGATGTCGATGATG | This study |
| *bipA*_qRT PCR_R | TTTAACGCGGTGGGTATCAAC | This study |
